# Exploring the comorbidity between personality and musculoskeletal disorders among adults: A scoping review

**DOI:** 10.3389/fpsyt.2022.1079106

**Published:** 2023-02-02

**Authors:** Shae E. Quirk, Heli Koivumaa-Honkanen, Bianca E. Kavanagh, Risto J. Honkanen, Jeremi Heikkinen, Lana J. Williams

**Affiliations:** ^1^School of Medicine, Institute for Mental and Physical Health and Clinical Translation, Deakin University, Geelong, VIC, Australia; ^2^Institute of Clinical Medicine, Psychiatry, University of Eastern Finland, Kuopio, Finland; ^3^Kuopio Musculoskeletal Research Unit (KMRU), Institute of Clinical Medicine, University of Eastern Finland, Kuopio, Finland; ^4^Mental Health and Wellbeing Center, Kuopio University Hospital, Kuopio, Finland; ^5^Barwon Health, University Hospital Geelong, Geelong, VIC, Australia

**Keywords:** personality disorder, personality disorder (MeSH), comorbidity, comorbidity [MeSH], musculoskeletal, musculoskeletal diseases, scoping review, review

## Abstract

**Introduction:**

There is growing awareness of the comorbidity between mental and musculoskeletal disorders (MSDs) and their associated burden. We aimed to explore what is known regarding the existing epidemiological clinical–and population– based literature on the comorbidity between personality disorders (PDs) and MSDs specifically. In addition, we aimed to investigate their associated burden by examining a range of outcomes including morbidity/mortality, patient- and clinical-reported outcomes, work-related outcomes, hospital admissions, and financial costs. Finally, we sought to identify gaps in the literature and provide recommendations for further research.

**Methods:**

Studies with participants 15 years of age were eligible. Categorical PDs/features (DSM-III/IV/5 or ICD 9/10), identified by a health care professional, medical records, diagnostic interviews, or self-administered questionnaires. The definitions/groupings of MSDs were guided by the ICD-10 including conditions of the back, joints, and soft tissue, and disorders of bone density and structure. Published peer-reviewed and gray literature were considered. Eligible study designs were cohort, case-control, and cross-sectional studies, and existing reviews of observational studies. Identification and selection of articles, data extraction and the presentation of the results was conducted according to the Joanna Briggs Institute methodological guidance and the PRISMA extension for scoping reviews.

**Results:**

In total, 57 articles were eligible including 10 reviews and 47 individual studies. Across clinical and population settings, we detected evidence of comorbidity between PDs and chronic back/neck/spine conditions, arthritis, and fibromyalgia, and emerging evidence of associations between PDs and reduced bone mineral density. In terms of knowledge gaps, the burden associated with PDs and MSDs is poorly understood, as is their underlying mechanisms.

**Discussion:**

This scoping review might prompt further research into PDs and MSDs as separate groups of disorders, along with their comorbidity and the mechanisms that may link them.

**Systematic review registration:**

https://osf.io/mxbr2/registrations.

## 1. Introduction

There is growing awareness of the comorbidity between mental and musculoskeletal disorders (MSDs) and their associated burden (1). Separately, mental disorders and MSDs are prevalent across the life course and are the leading contributors to disability worldwide (2, 3). Together, they account for just over one third (33.9%) of the global years lived with disability (YLDs) (1, 4). Thus far, there has been no broad-level exploration or synthesis of the comorbidity between personality disorders (PDs) specifically and MSDs.

Taking into account methodological differences—approximately one in eight people in Western countries have a form of PD (5)—the worldwide pooled prevalence is estimated to be 7.8% [95% confidence interval (95%CI), 6.1–9.50] (6). With an often-earlier age of onset between childhood and adulthood (7), PD is a term used to describe patterns of symptoms, behaviors, and experiences that can be inflexible, enduring, and impairing (see Supplementary Box 1) and whereby personality structure presents difficulties for developing adaptive solutions to universal life tasks (7). People with PDs or features of these mental disorders often have difficulty regulating emotions and may use maladaptive ways of coping to inhibit or modulate distressing/painful feelings or thoughts. These experiences can lead to disrupted adaptive functioning including forming and maintaining a stable sense of self and relationships with peers, partners, and family members, work and school, and good self-care (8, 9). In addition, the physical health of people with PDs is of growing concern. PDs are associated with health risk factors including heavier weight/obesity (10–12), physical disability linked to substance use (13), and barriers to quality mental and physical healthcare (14, 15), especially among younger people (14), and broad physical health conditions (10–12).

Separately, the World Health Organization (WHO) defines MSDs as a group of conditions that include approximately 150 discrete International Classification of Diseases (ICD) diagnoses (16). MSDs affect bones, joints, muscles and other soft tissues—ranging from acute onset with short duration to the chronic and disabling (16). The most common forms of MSDs are frequently characterized by pain and restricted mobility, and include conditions of the back or spine (e.g., chronic back or neck pain), joint diseases (e.g., types of arthritis), disorders of bone density and structure (e.g., osteopenia and osteoporosis), and soft tissue diseases [e.g., muscular pain/myalgia or fibromyalgia (see Supplementary Box 2) (16). The burden and consequences associated with MSDs are significant, including increased risk of other chronic diseases (17).

Using a biopsychosocial model, conceptually, the comorbidity of PDs and MSDs may be linked via several pathways. Much research has linked PD and types of chronic pain which is suggested to be in part, due to self-regulatory difficulties among some patients and increased vulnerability/sensitivity to physical pain (18–22). However, the extent to which MSDs may be an underlying cause of chronic pain is not well understood. Among people with PDs and MSDs, the dynamic nature of psychosocial stressors and physical pathology may modulate one’s perception/experience of their health and symptoms, and the capacity to cope—potentially maintaining or worsening symptoms (21, 23–25).

A preliminary search of Google Scholar, Medline Complete, PROSPERO, PubMed, the Cochrane Database of Systematic Reviews, JBI Evidence Synthesis, and Open Registries was conducted, and no current or underway systematic or scoping reviews on the topic were identified. We identified several narrative/descriptive reviews that reported on published articles on PD and a broad range of physical comorbidities, which also explored potential underlying mechanisms (18, 19, 26–32). However, no existing review performed a synthesis of evidence on the comorbidity between PD and the full range of MSDs.

Therefore, the objectives of this review were to explore and understand the extent and type of evidence on the comorbidity of PDs and MSD among people aged ≥ 15 years, and the burden associated with their comorbidity in clinical and population-based settings. For this review, comorbidity refers to having both a PD and MSD. In addition, we aimed to identify knowledge gaps on this topic and propose recommendations for future research.

The research questions were:

• What is known from the existing clinical– and population– based literature regarding the comorbidity between PDs and MSDs?
• What is known from the existing literature regarding disease burden associated with the comorbidity between PDs and MSDs?
• What are the knowledge gaps in relation to this topic?
• What recommendations for future research, including systematic reviews, can be made?

Given our objectives, a scoping review methodology was identified to be the most appropriate approach (33).

## 2. Methodology

The protocol for this study was guided by Arksey and O’Malley’s methodological framework for scoping studies (34), a published protocol (35), the most recent guidance published from the JBI (33, 36), and the Preferred Reporting Items for Systematic Reviews extension for Scoping Reviews (PRISMA-ScR) (36).

### 2.1. Eligibility criteria

The authors developed eligibility criteria using the ‘Population–Concept–Context (PCC)’ framework recommended by JBI for scoping reviews (37).

### 2.2. Participants

Given PD often emerges earlier in life —and to ensure that potentially relevant studies were identified that may utilize age-stratified samples—studies with participants aged ≥ 15 years were considered eligible. Other than age, there were no specific exclusions based on any participant characteristics. In addition, studies were considered if they examined people with categorical PDs and features of PDs according to the Diagnostic and Statistical Manual of Mental Disorders (DSM-III/IV/5) or ICD 9/10, identified by a relevant health professional, medical record, diagnostic interviews or self-administered questionnaires/self-reports. As such, trait models of personality in relation to MSDs were beyond the scope of the current review.

### 2.3. Concept

The comorbidity between PDs and MSDs was the primary concept for this review. In order to yield a wide scope of literature, a broad definition of MSDs was adapted from the WHO, including conditions that affect joints, bones, muscles, spine, and multiple body areas (16). The definitions and groupings of MSDs were further refined and guided by the ICD-10 (38). These included: conditions of the back (M40–M54), conditions of the joints (M00–M25), soft tissue conditions (M60–M79), disorders of bone density and structure (M80–M94), and “other” (e.g., studies that examine MSDs as a group or make comparisons between different MSD groups). Therefore, types of non-MSD-related chronic pain in relation to PD were out of the scope of this review.

Studies that examined or included measures of burden in relation to the comorbidity between PDs and MSDs were eligible including: morbidity, patient-reported outcomes, clinician-reported outcomes, work-related outcomes, hospital admissions, mortality, financial costs, other indicators such as disability adjusted life years (DALY), quality adjusted life years (QALYs), or YLDs. Unintentional injuries and falls were beyond the scope of the current review.

### 2.4. Context/Settings

Studies worldwide were considered eligible if they were from either population-based or clinical settings.

### 2.5. Types of sources

This scoping review considered a wide range of evidence sources including published peer-reviewed and published grey literature. Observational studies (analytical/descriptive) including cohort, case-control, and cross-sectional studies, and existing reviews of observational studies were eligible. For this review, published gray literature was considered pertinent sources of epidemiological evidence. Eligible grey literature included published dissertations. We also considered published reports utilizing epidemiological data from government agencies and their relevant departments as pertinent sources of information, due to the capability to inform public health planning/policies and clinical practice.

### 2.6. Exclusion criteria

Studies were excluded if they:

• Were not published in English.
• Were correspondences, letters, opinion papers or qualitative studies (including reviews of qualitative studies).
• Did not assess PDs according to the eligibility criteria.
• Did not examine MSDs according to the eligibility criteria.
• Examined populations aged < 15 years.

### 2.7. Study identification and selection

A comprehensive search strategy was developed to identify published peer-reviewed studies, and gray literature (see section 2.5 Types of sources). The history of the search strategy during the protocol development phase is previously published (35).

The text words contained in the titles and abstracts of relevant articles, and the index terms or keywords were used to develop a complete search strategy for Medline Complete, CINAHL, and PsycINFO via the EbscoHost platform. The search strategy, including all identified keywords and index terms, were appropriately translated for each database (see Supplementary Table 1).

The search strategy was reviewed and evaluated by a medical librarian (BK) using the Peer Review of Electronic Search (PRESS) checklist (39). It was implemented on 7 September 2020 by one reviewer (SEQ); no language or date restrictions were applied. In addition, a list of articles (32, 40–45) was compiled and cross-checked in the search results, to ensure the appropriate literature was targeted and sourced. The list of articles was selected based on the authors’ existing knowledge of the literature, and from the conduct of a prior review (31). To identify further potentially relevant published articles, the reference lists of all included review studies were screened. Sources of published gray literature and/or additional published articles were searched using an adapted search in Google (advance search). It was predetermined that all pages of the Google search results would be screened by one reviewer. The results were narrowed by the find pages “with all these words” search option and by file type (PDF/documents). Records identified as potentially relevant were then assessed according to the eligibility criteria, and the whole review team agreed on their inclusion.

**FIGURE 1:**
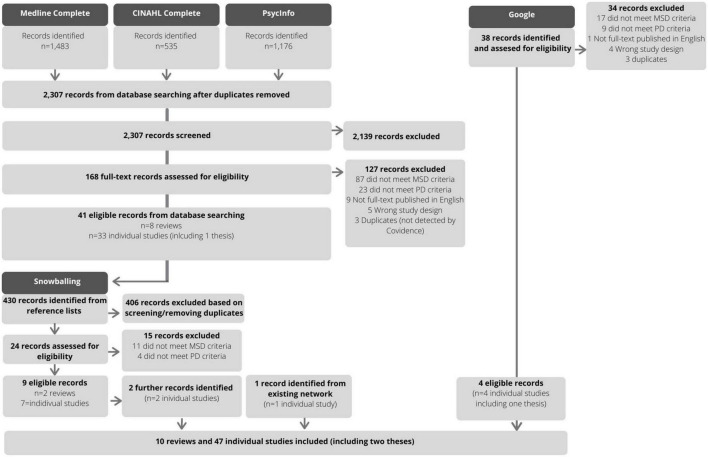
Flow diagram of study identification and selection.

Two reviewers (SEQ and BEK) pilot tested a screening tool on a random selection of citations from the database search (*n* = 25), then discussed the findings with the entire team. The same reviewers then independently screened titles and abstracts, and a consensus meeting was held between the reviewers and the supervising author to discuss discrepancies, which were not common (5% conflicts). The reviewers then completed full-text reviews, independently, with conflicts (16%) resolved in one consensus meeting. To identify further sources, one reviewer (SEQ) searched and screened the reference lists of eligible reviews. Where more detail was required, the abstracts or full-text articles were sourced. The results of the search and reasons for exclusion at the full-text review stage are presented in Figure 1.

### 2.8. Data management and extraction

All identified citations were collated and uploaded into Mendeley and Covidence, with duplicates removed. The whole review team developed, then two reviewers’ independently pilot tested a charting form on a sample of three studies (see Supplementary Table 2). In line with published guidance, critical appraisal of the included studies was not performed (33).

### 2.9. Synthesis of results

We intended to scope a range of literature, and as a result, we yielded a wide range of study designs, populations, and settings. Therefore, our approach to the synthesis was intentionally descriptive—providing readers with an overview of the research and findings conducted in this field to date rather than a systematic review or meta-analysis. The results of the search strategy and selection process are presented in a flow diagram (see Figure 1). The characteristics of individual studies are presented in a table according to study population, setting, and design (see Supplementary Table 3). The main results are presented according to the research questions (in text) and in tables (see Tables 1, 2).

**TABLE 1:** Summary of relevant findings on the comorbidity between PD and MSDs, according to MSD category, study population, and citation.

Citation country (study design)	Study population; sample size (*n*) Sex:% female	Mean age (SD)/median (IQR)/age range	PD assessment	MSD assessment	Summary of relevant findings
**Conditions of the back**					
**Clinical studies reporting on the comorbidity of personality disorder among patients with conditions of the back**					
Dersh et al. (57) USA (Cross-sectional)	Patients entering the PRIDE functional restoration program N: 1,323 Sex: 38.3% female	41.9 (9.6)	DSM-IV SCID-II (expert)	Grouped spinal disorders according to pain/injury site: cervical and/or thoracic, lumbar, and cervical/thoracic and lumbar (expert diagnosis)	• 69.6% of patients with spine disorders had a PD • The frequency of specifics PDs were: 30.8% paranoid; 2.6% schizoid; 4.5% schizotypal; 4.5%; antisocial; 27.9% borderline; 17.3% histrionic; 13.8% narcissistic; 12.7% avoidant; 7.3% dependent; 15.9% obsessive-compulsive; 16.6% personality disorder NOS
Fishbain et al. (59) USA (Cross-sectional)	Chronic pain patients attending the University of Miami Comprehensive Pain Center N: 221 Sex: 42% female	41.1 (10.0)	DSM flowcharts/clinical impression	Chronic low back pain (presenting problem to pain centre)	• More patients with low back pain who were “smokers” had histrionic PD (61.7%) compared to “non-smokers” (38.3%) patients [χ^2^16.1 (1), p = 0.001] • More “non-smokers” had obsessive-compulsive PD with 77.2% compared to “smokers” with 22.8% [χ^2^(1) = 15.4, *p* = 0.001] • 35.1.% of “smokers” and 64.9% of “non-smokers” had dependent PD (ns)
Long et al. (75) USA (Cross-sectional)	Patients who were treated for chronic back pain at the Johns Hopkins Pain Treatment Program N: 78 Sex: 66.1% female	19–67	DSM-III Clinical impression/collateral sources	Chronic low back (expert diagnosis/review of medical records)	• 43.6% of patients with chronic low back pain had a probable PD
Polatin et al. (80) USA (Cross-sectional)	Patients entering the PRIDE functional restoration program N: 200 Sex: 33% female	nr	DSM-III-R SCID-II (expert)	Chronic low back pain (expert diagnosis)	• 51% patients with chronic low back pain had a PD; 21% had one PD and 30% had two or more PDs • The frequency of specifics PDs were: 33% paranoid; 4% schizoid; 4% schizotypal; 5% antisocial; 15% borderline; 4% histrionic; 5% narcissistic; 14% avoidant; 3% dependent; 6% obsessive-compulsive; 2% personality disorder NOS; passive-aggressive 12%; self-defeating 10%
**Clinical studies reporting on the comorbidity of conditions of the back among patients with personality disorder**					
Frankenburg and Zanarini (11) USA (Prospective cohort)	Patients enrolled in the MSAD study N: 264 (total) N: 74 (borderline PD with obesity) N: 190 (borderline PD without obesity) Sex: 87.8% female (borderline PD with obesity) Sex: 77.9% female (borderline PD without obesity)	Borderline PD with obesity 35.0 (6.1) Borderline PD without obesity: 32.2 (5.6)	DSM-III-R DIB-R (expert)	Chronic back pain (expert diagnosis)	• 44.3% patients with borderline PD had chronic back pain • 58.1% and 39.0% of patients with and without obesity had chronic back pain at the 6-year follow-up [RR 1.5 (95% CI 1.15–2.10)]
Braden and Sullivan (53) USA (Cross-sectional)	Community-based respondents enrolled in the NCS-R N: 5,692 Sex: 58.6% female (with lifetime self-reported pain) Sex: 46.6% female (without lifetime self-reported pain	Aged 18 +	IPDE Screener	Chronic back/neck problems (self-reported)	• 27.2% of people with chronic neck/back pain screened positive for borderline/antisocial PD (grouped) • More people with “other” chronic pain screened positive for borderline/antisocial PD compared to people with chronic neck/back pain [36.3% vs. 27.2%; χ^2^(1) = 14.19, *p* < 0.001]
Gerhardt et al. (60) Germany (Cross-sectional)	Population-based respondents of a postal survey of back pain by the GBPRN N: 110 Sex: 57% female	18–74 years	DSM-IV SCID-II (nr)	Chronic back pain (self-report/expert verified)	• 15.5% of people with chronic back pain had any PD • Cluster C PDs were the most common with avoidant and obsessive-compulsive PDs (4.5% each), then borderline PD (3.6%), paranoid PD (2.7%), and narcissistic (PD) 0.9%
McWilliams and Higgins (77) USA (Cross-sectional)	Community-based respondents enrolled in Part II of the NCS-R N: 5,692 Sex: nr	Aged 18 +	ICD-10 Adapted IPDE screener using borderline PD items (self-report)	Spinal pain (self-report)	• People with past-year spinal pain had higher mean IPDE screen (e.g., borderline PD symptoms) item scores for borderline PD [M = 2.04 (SE = 0.08)] compared to those with lifetime/remitted spinal pain [M = 1.73 (SE = 0.08)], and those without any history [M = 1.38 (SE = 0.04), *p* < 0.01)] • In further analyses, compared to people with no history, people with past year spinal pain (*b* = 0.38, *p* < 0.01), or remitted spinal pain (*b* = 0.31, *p* < 0.01) had higher borderline PD symptoms (adjusted for sociodemographic variables and past-year mood, anxiety, and externalizing disorders)
**Conditions of the joints**					
**Clinical studies reporting on the comorbidity of personality disorder among patients with conditions of the joints**					
Marcenaro et al. (73) Italy (Cross-sectional)	In- and outpatients receiving treatment at a rheumatology department N: 15 Sex: nr	54 (12.8)	DSM-III-R SCID-II (nr)	Rheumatoid arthritis (expert diagnosis)	• 87% of patients with rheumatoid arthritis had PD
**Clinical studies reporting on the comorbidity of conditions of the joints among patients with personality disorder**					
Frankenburg and Zanarini (11) USA (Prospective cohort)	Patients enrolled in the MSAD study N: 264 (total) N: 74 (borderline PD with obesity) N: 190 (borderline PD without obesity) Sex: 87.8% female (borderline PD with obesity) Sex: 77.9% female (borderline PD without obesity)	Borderline PD with obesity 35.0 (6.1) Borderline PD without obesity: 32.2 (5.6)	DSM-III-R DIB-R (expert)	Osteoarthritis (expert diagnosis)	• 9.8% patients with borderline PD had osteoarthritis • 24.3% and 4.2% of patients with and without obesity had osteoarthritis at the 6-year follow-up [RR = 5.8 (95%CI, 2.63–12.71)]
Sansone et al. (25) USA (Cross-sectional)	Admission to a sub-acute detoxification unit for opioid dependence, in which buprenorphine is the standardised treatment N: 111 Sex: 46.5% female	18 to 59 years (M-32.80, SD-9.04)	DSM-IV PDQ-4 (self-report)	Rheumatoid arthritis (self-report)	• PDQ scores were not significantly associated with rheumatoid arthritis among patients with opioid dependence
**Population-based studies reporting on the comorbidity of personality disorder and conditions of the joints**					
El-Gabalawy et al. (22) USA (Cross-sectional)	Wave 2 NESARC participants N: 34,653 Sex: 52.1% female	Aged 20 +	DSM-IV AUDADIS-IV (lay interviewer)	Arthritis (self-report)	• 27.7% and 21.4% of people with and without borderline PD had arthritis, respectively. • People with borderline PD had increased odds of arthritis [OR = 1.56 (95%CI, 1.31–1.85)] • Analyses adjusted for sociodemographic factors, any anxiety, mood, or substance use disorder, and other PDs
El-Gabalawy et al. (82) USA (Prospective cohort)	Wave 1 and 2 NESARC participants aged 55 + N: 10,409 Sex: 55.4% female	Aged 55 +	DSM-IV AUDADIS-IV (lay interviewer)	Arthritis (self-report)	• PD did not significantly predict incident arthritis among people aged 55 + years with anxiety disorder
Goldstein et al., (50) USA (Cross-sectional)	Wave 1 NESARC participants N: 43,093 Sex: nr	48 (13.3)	DSM-IV AUDADIS-IV (lay interviewer)	Arthritis (self-report)	• For men, the comorbidity of arthritis was: 18.2% for antisocial PD, 14.2% for antisocial features, 12.6% for conduct only, and 12.4% without any history • For women, the comorbidity of arthritis was: 22.6% for antisocial PD, 18.5% for antisocial features, 11.0% for conduct only, and 21.0% without any history • Men [OR = 2.2 (95%CI, 1.69–2.76)] and women [OR = 1.4 (95%CI, 1.03–1.96)] with antisocial PD had increased odds of arthritis compared to men and women without a history, respectively • Analyses adjusted for sociodemographic factors, past-year personal income, health insurance coverage, region and urbanicity, health risk factors, lifetime nicotine dependence, mood, anxiety, any alcohol use, and substance use disorders, other PDs, pathological gambling, and any additional PDs
McWilliams et al. (76) USA (Cross-sectional)	Wave 1 NESARC participants N: 43,093 Sex: nr	Aged 18 +	DSM-IV AUDADIS-IV (lay interviewer)	Arthritis (self-report)	• The frequencies for comorbid specific PDs among people with arthritis were: 5.6% paranoid, 4.7% schizoid, 2.2% histrionic, 4.1% antisocial, 3.5% avoidant, and 10.3% obsessive-compulsive PDs, and 0.9% for dependent PD • Compared to without, people with arthritis had increased odds of paranoid [OR = 1.40 (95%CI, 1.17–1.67)], schizoid [OR = 1.79 (95%CI, 1.48–2.17)], histrionic [OR = 1.80 (95%CI, 1.36–2.39)], antisocial [OR = 2.06 (95%CI, 1.72–2.48)], avoidant [OR = 1.62 (95%CI, 1.27–2.06)], and obsessive-compulsive [OR = 1.41 (95%CI, 1.23–1.62)] PDs (all < *p* = 0.05), but not dependent PD • Analyses were adjusted for sex, marital status, income, age, past-year anxiety, depressive, substance use disorders, ≥ 1 other health condition
McWilliams and Higgins (77) USA (Cross-sectional)	Community-based respondents enrolled in Part II of the NCS-R N: 5,692 Sex: nr	Aged 18 +	ICD-10 Adapted IPDE screener using borderline PD items (self-report)	Arthritis (self-report)	• People with past-year arthritis tended to have higher mean IPDE screen (e.g. borderline PD symptoms) item scores for borderline PD [M = 1.61 (SE = 0.07)] compared to those without any history [M = 1.52 (SE = 0.03), but the results did not reach statistical significance • In further analyses, compared to people with no history, people with past year arthritis (*b* = 0.19, p < 0.01) had higher borderline PD symptoms (adjusted for sociodemographic variables and past-year mood, anxiety, and externalizing disorders)
Powers and Oltmanns (43) USA (Cross-sectional)	Community-based residents aged 55–64 years enrolled in the SPAN N: 1,051 Sex: 53% female	59.4 (2.7)	DSM-IV SIDP-IV (trained interviewers)	Arthritis (self-reported)	• Compared to without, adults aged 55–64 years with borderline PD were more likely to have arthritis [OR = 2.64 (95%CI, 1.06–6.57)] • Analyses adjusted for sociodemographic, and lifetime mental disorders including other PDs • The association was fully mediated by BMI
Quirk et al. (32) USA (Cross-sectional)	Wave I and 2 NESARC participants N: 34,653 Sex: 52.1% female	Aged 20 +	DSM-IV AUDADIS-IV (lay interviewer)	Arthritis (self-report)	• 27.2% of people with PD compared to 21.4% without had arthritis • In further analyses, the odds for arthritis differed among younger (< 55 years) [OR = 1.36 (95%CI, 1.13–1.64), *p* < 0.001] and older adults (≥ 55 years) [OR = 1.22 (95%CI, 1.03–1.43), *p* = 0.01] with any PD • People < 55 years with schizoid PD had the highest odds of arthritis [OR = 1.62 (95%CI, 1.16–2.26), *p* < 0.001] • Analyses were adjusted for sociodemographic factors and past year mood, anxiety, and substance use disorders
Quirk et al. (31) Australia (Cross-sectional)	Community-based women enrolled in the GOS in south-eastern Australia N: 765 Sex: 100% female	56.8 (42.7–68.9	DSM-5 SCID-II (trained interviewer)	Arthritis (self-reported)	• 30.13% women with and 32.0% without any PD had arthritis • In further analyses, compared to without, women with Cluster B PD [OR = 4.25 (95%CI, 1.34-13.44) had higher odds of arthritis • Analyses were adjusted for sociodemographic and lifestyle factors and other mental disorders
**Soft tissue conditions**					
**Clinical studies reporting on the comorbidity of personality disorder among patients with soft tissue conditions**					
Fu et al. (47) USA (Cross-sectional)	Patients attending an outpatient rheumatology office N: 48 Sex: 95.8% female	49.3 (nr)	DSM-IV PDQ-4 (self-report)	Rheumatology department record review fibromyalgia according to ACR criteria	• 56.3% of patients with fibromyalgia had a possible PD including avoidant (27.1%), depressive (25.0%), paranoid (22.9%), and obsessive- compulsive (20.8%) PDs
Gumà-Uriel et al. (69) Spain (Cross-sectional)	Patients enrolled in the FibroQoL study, a psychoeducational program for fibromyalgia N: 157 Sex: 98.1%	18–75	DSM-IV IPDE Screener (self-report)	Identified patients with fibromyalgia according to ACR criteria (database/records)	• 65.0% of patients with fibromyalgia had a possible PD • Of those with a PD, Cluster C PDs were the two most common including avoidant PD (41.4%) and obsessive-compulsive PD (33.1%) and then borderline PD (27.0%).
Thieme et al. (61) Germany (Cross-sectional)	Patients attending a rheumatologic outpatient department and Hospital for Rheumatic Disorders at Berlin-Buch N: 115 Sex: 100% female	48.17 (10.32)	DSM-IV SCID-II (expert)	Fibromyalgia according to ACR criteria (expert diagnosis)	• 8.7% of patients with fibromyalgia had PD
Uguz et al. (66) Turkey (Case-control)	Patients attending Rheumatology Outpatient Clinic at a University hospital N: 103 cases N: 83 controls Sex: nr	nr	DSM-III-R SCID-II (expert)	Fibromyalgia according to ACR criteria (expert diagnosis)	• Patients with fibromyalgia had a higher percentage of PD with 31.1% vs. 13.3% (control); avoidant PD 10.7% (patient) with vs. 2.4% (control); and obsessive-compulsive PD 23.3% (patient) vs. 3.6% (control); all < *p* < 0.05
Kayhan et al. (64) Turkey (Case-control)	Patients with fibromyalgia attending the Outpatient Physical Therapy Unit of Mevlana University N: 190 Patient group: 96 Healthy group: 94 Sex: 100% female	37.75 (6.24) Patient: 38.27 (6.18) Healthy: 37.23 (6.29)	DSM-IV SCID-II (expert)	Fibromyalgia according to ACR criteria (expert diagnosis)	• 13.5% of patients with fibromyalgia had PD vs. 5.3% controls • The frequency of other PDs was low: avoidant PD 2.1% (patient) VS. 0.0% (healthy); dependent PD 2.1% (patient) vs. 1.1% (healthy); and obsessive-compulsive PD 1.0% (patient) vs. 2.1% (healthy)
**Clinical studies reporting on the comorbidity of soft tissue conditions among patients with personality disorder**					
Sansone et al. (22) USA (Cross-sectional)	Admission to a sub-acute detoxification unit for opioid dependence, in which buprenorphine is the standardized treatment N: 111 Sex: 46.5% female	18 to 59 years (M-32.80, SD-9.04)	DSM-IV PDQ-4 (self-report)	Fibromyalgia (self-report)	• PDQ scores were not significantly associated with self-reported fibromyalgia among buprenorphine patients
**Population-based studies reporting on the comorbidity of personality disorder and soft tissue conditions**					
Olssøn and Dahl (67) Norway (Case-control)	Community-based respondents to the HUBRO study health Survey N: 2,214 Cases: 369 Controls: 1,845 Sex: 48% female	Aged 30 +	DSM-IV IPDS	Fibromyalgia (self-reported)	• Slightly more people who screened positive for PD reported having fibromyalgia with 4% vs. 2% who screened negative (controls) (*p* = 0.04)
Olsson and Dahl (68) Norway (Case-control)	Community-based respondents to the HUBRO study health Survey Cases:280 Controls: 1,400 Sex: 65% female	Aged 30 +	DSM-IV Avoidant PD items of the IPDS	Muscular pain (self-reported)	• More people who screened positive for avoidant PD reported having muscular pain with 37% vs. 20% who screen negative (controls) • In univariate associations, people who screened positive for avoidant PD had increased odds of muscular pain [OR 2.37 (95% CI 1.80-3.13, *p* < 0.001) • In multivariate analyses, the association was no longer statistically significant (variables relating to sociodemographic, and mental and somatic impairments)
Russek et al. (78) USA (Cross-sectional)	Survey respondents accessing the National Fibromyalgia Association website N: 1,125 Sex: 97.6% female	Median range 40–49	DSM-IV Self-report questionnaire based on criteria for OCPD	Fibromyalgia (self-reported)	• 26.8% of people with fibromyalgia had possible obsessive-compulsive PD
**Disorders of bone density and structure**					
**Clinical studies reporting on the comorbidity of disorders of bone density and structure among patients with personality disorder**					
Kahl et al. (45) USA (Cross-sectional)	Patients attending a Specialized unit for the treatment of borderline PD N: 38 (total) N: 16 (borderline PD alone) N: 12 (borderline PD + ever MDD) N: 10 (borderline PD + current MDD) Sex: 100% female	Borderline PD alone: 25.9 (5.0) Borderline PD + MDD: 31.8 (6.5)	DSM-IV SCID-II (expert)	BMD measured using dual-energy X-ray absorptiometry at the lumbar spine, right femur, left femur, and the forearm of the non-dominant hand Osteopenia defined as a T-score ≤ –1	• Bone mineral density was lower at the lumbar spine for patients with borderline PD plus a MDD than patients with borderline PD alone (*p* < 0.05) • Osteopenia at the lumbar spine was present in 20% of patients with borderline PD plus MDD compared to 6% of patients with borderline PD alone • Analyses were age-weight adjusted
Kahl et al. (41) USA (Cross-sectional)	Patients attending a Specialized unit for the treatment of borderline PD N: 12 (MDD30) N: 12 (MDD43) N: 23 (borderline PD + MDD N: 16 (borderline PD alone) Sex: 100% female	MDD: 20–51 years; MDD30: 30 MDD43: 42.9 Borderline PD + MDD: 18–43 years; Borderline PD alone: 19-34	DSM-IV SCID-II (expert)	BMD measured using dual-energy X-ray absorptiometry at the lumbar spine, right femur, left femur, and the forearm of the non-dominant hand Osteopenia defined as a T-score ≤ –1	• Women with comorbid borderline PD and MDD had lower bone mineral density at the lumbar spine than women in the MDD30 (mean age 30 years) and borderline PD alone groups (all *p* < 0.05). • The frequency of osteopenia at the lumbar spine in order was: MDD43 (mean age 43 years) 33%, comorbid borderline PD and MDD 9%, MDD30 8%, and borderline PD alone 6% • Analyses were age-weight adjusted
**Population based studies reporting on the comorbidity of personality disorder and disorders of bone density and structure**					
Williams et al. (63) Australia (Cross-sectional)	Community-based women enrolled in the GOS in south-eastern Australia (2011-2014) N: 696 Sex: 100% female	56.8 (42.7–68.9)	DSM-5 SCID-II (trained interviewer)	Bone mineral density [Areal BMD (g/cm2)] was measured at the posterior–anterior (PA) spine (L2–4), femoral neck (hip), and total body including head using dual-energy X-ray absorptiometry Osteoporosis was determined by a BMD T-score of < -2.5	• Compared to women without, women with Cluster A PD had lower hip bone mineral density (*p* < 0.05) • No statistically significant associations between women with Cluster B and C PDs with bone mineral density • No significant difference between women with or without PD and comorbid osteoporosis (6.1% vs. 8.7%) • Analyses were age-weight adjusted
**Other**					
Dersh et al. (56) USA (Cross-sectional)	Patients entering the PRIDE functional restoration program N: 1,595 Sex: 41.9% female	42.1 (9.6)	DSM-IV SCID-II (expert)	Grouped musculoskeletal/spina disorders grouped according to pain/injury site: lumbar spine, cervical spine, multiple spine areas, upper extremity neuropathic, upper extremity non-neuropathic, and three or more (polymorphous) musculoskeletal areas (expert diagnosis)	• 70.0% of patients with MSDs had a PD • The percentage of specific PDs among MSD patients were: paranoid PD 31.0%, schizoid PD 2.6%, schizotypal 4.8%, antisocial PD 4.3%, borderline PD 27.5%, histrionic PD 17.8%, narcissistic PD 13.8%, avoidant PD 12.9%, dependent PD 7.3%, and obsessive-compulsive PD 16.3%.
Howard (51) USA (Cross-sectional)	Patients entering the PRIDE functional restoration program N: 3,492 Sex: *Varies depending on subgroup examined	*Varies depending on subgroup examined	DSM-IV SCID-II (expert)	Grouped musculoskeletal/spina disorders grouped according to pain/injury site: lumbar spine, cervical spine, multiple spine areas, upper extremity neuropathic, upper extremity non-neuropathic, and three or more (polymorphous) musculoskeletal areas (expert diagnosis)	• The frequency of PD did not statistically differ according to different musculoskeletal region/site involved in the pain/condition
Linder et al. (72) Sweden (Cross-sectional)	Patients referred by an insurance office to the Diagnostic Centre at the Karolinska Hospital who were long-term sick leavers N: 416 Fibromyalgia: 92 Myalgia group: 44 Spine/joints: 111 Depression: 169 Sex: 100% female	Fibromyalgia: 45.6 (10.2) Myalgia: 44.4 (8.1) Spine/joints: 46.4 (8.2) Depression: 46.5 (9.5)	DSM-IV SCID-II (expert)	Fibromyalgia, myalgia, and diseases of spine/joints according to ICD-10 criteria (expert diagnosis)	• Patients with MSDs who were long-term “sick leavers” had mean sum PD criteria scores below diagnostic thresholds
López-Ruiz et al. (70) Spain (Case-control)	Patients attending the Rheumatology Departments of the Hospital del Mar and Hospital CIMA-Sanitas in Barcelona OA-CS group: 19 OA-noCS group: 41 Fibromyalgia group: 47 Control group: 26 Sex: 84.2% female (OA-CS) Sex: 65.9% female (OA-noCS) Sex: 100% female (fibromyalgia) Sex: 59.3% female (control	OA-CS: 66.37 (8.77) OA-noCS: 66.8 (7.39) Fibromyalgia: 46.47 (7.92) Control: 51.56 (11.41)	DSM-IV MCMI-III (self-report)	Osteoarthritis (with and without CS) (expert diagnosis) Fibromyalgia according to ACR criteria (expert diagnosis)	• There was no significant association between clinically significant MCMI profiles across the MSD groups

ACR, American College of Rheumatology; AUDADIS-IV, alcohol use disorder and associated disabilities interview schedule-IV; BMD, bone mineral density; BMI, body mass index; CI, confidence interval; CS, central sensitization; DIB-R, diagnostic interview for borderlines-revised; DSM, diagnostic and statistical manual of mental disorders; Dx, diagnosis; GOS, Geelong osteoporosis study; HUBRO, The Oslo health study; ICD, international classification of diseases and related health problems; IPDE, international personality disorder examination; MCMI, Millon clinical multiaxial inventory; MDD, major depressive disorder; MSAD, McLean study of adult development; MSD, musculoskeletal disorders; NCS-R, National Comorbidity Survey-Revised; NESARC, National Epidemiological Survey on Alcohol and Related Conditions; nr, not reported; OR, odd ratio; OA, osteoarthritis; PD, personality disorder; PDQ-4, personality diagnostic questionnaire-4; PRIDE, Productive Rehabilitation Institute of Dallas for Ergonomics; QoL, quality of life; RR, relative risk; RRR, relative risk ratio; SCID-II, structured clinical interview for DSM Axis II personality disorders; SIDP-IV, structured interview for DSM-IV personality; SPAN, St. Louis personality and aging network.

**TABLE 2:** Summary of relevant findings on the burden associated with the comorbidity of PDs and MSDs, according to identified concepts and citation.

Citation country (study design)	Study population; sample size (*n*) Sex:% female	Mean age (SD)/median (IQR)/age range	PD assessment	MSD assessment	Concept of burden applied	Summary of relevant findings
**Morbidity**						
Breckenridge and Clark (54) USA (Retrospective cohort)	Patients attending the Stanford University and the Veterans Affairs Palo Alto Health Care System N: 200 N: 100 (“N” group; received (NSAIDs) N: 100 (“O” group received opioid drugs) Sex: 5% female (N) Sex: 6% female (O)	N: 61.8 (11.7) O: 61.5 (13.0)	ICD-9 Chart review	Chart review of grouped backache/lumbago, postlaminectomy syndrome/lumbosacral neuritis/lumbosacral spondylosis without myelopathy/displacement of lumbar disk/degeneration of lumbar or lumbosacral disk/lumbar spinal stenosis according to ICD-9 codes	Morbidity • Comorbidity • Opioid medication • NSAID medication use	• More MSD patients who were long-term opioid users had PD with 14% vs. 1% of patients using NSAIDs (*p* < 0.001) • Compared to the NSAID use group, patients with MSDs and comorbid PD were more likely to belong to the opioid use group [OR = 18.61 (95%CI, 1.54–224.09), *p* < 0.02] • Analyses adjusted for sociodemographic factors, psychiatric diagnoses other than the predictor, and treatment utilisation factors.
Campbell et al. (62) Australia (Cross-sectional)	Participants with chronic non-cancer pain enrolled in the POINT study recruited through community pharmacies N: 978 Sex: 55.3% female	57.5 (13.6)	ICD-10 Adapted IPDE screener using borderline PD items (self-report)	Arthritis, chronic back/neck pain, and fibromyalgia (self-report)	Morbidity • Comorbidity • Benzodiazepine use • Accidental overdose • Opioid dependence	• 19.1% of people in the community who were prescribed opioids for pain had comorbid positive screen for borderline PD • Compared to without, people with borderline PD positive screen were more likely to report a past-year chronic back/neck condition [OR = 1.55 (95%CI, 1.02–2.37), *p* = 0.04], fibromyalgia [OR = 1.94 (95%CI, 1.18–3.15), *p* = 0.008], higher oral morphine equivalent [mg/day; M = 101.7 (range = 50–180), *p* < 0.001], daily benzodiazepine use [OR 2.30 (95%CI, 1.59–3.32, *p* < 0.001), and accidental overdose [OR 3.47 (95%CI, 1.59–7.77), *p* = 0.03] • In further analyses—adjusting for sociodemographic factors, pain-related factors, mental health symptoms, and lifetime alcohol/drug use disorder—people with borderline PD positive screen had greater odds of lifetime opioid dependence [OR = 2.52 (95%CI, 1.43–4.47, *p* = 0.002]
Frankenburg et al. (46) USA (Prospective cohort)	Patients enrolled in the MSAD study N: 264 Sex: 80.7% female	33.0 (SD = 5.8)	DSM-III-R DIB-R (expert)	Osteoarthritis, back pain, and fibromyalgia (expert diagnosis)	Morbidity • Comorbidity • Opioid medication use	• Comorbid chronic back pain [OR = 1.95 (95%CI, 1.41–2.70), fibromyalgia [OR = 3.29 (95%CI, 1.70–6.36), and osteoarthritis [(OR = 3.32 (95%CI, 2.08–5.29)] were predictors of opioid medication use among patients with borderline PD after 10-years follow-up • Analyses were adjusted for (other than the predictor) time-varying back pain, osteoarthritis, fibromyalgia, and baseline history of drug abuse/dependence
**Patient-reported outcomes**						
Brede et al. (55) USA (Cross-sectional)	Patients entering the PRIDE functional restoration program N: 551 Sex: 52% female	47.2 (9.9)	DSM-IV SCID-II (expert)	Grouped musculoskeletal disorders involving pain/injury of cervical/thoracic/lumbar extremity/multiple spinal/multiple musculoskeletal with at least one spinal (expert diagnosis)	Patient-reported outcome • Symptoms of pain anxiety according to the Pain Anxiety Symptom Scale	• Among patients with MSDs, a “dose response” type-pattern of PD frequency was observed according pain anxiety symptoms scales scores: 40%, 52%, and 65% patients with low, medium, and high pain anxiety symptom scores (*p* < 0.001)
Gumà-Uriel et al. (69) Spain (Cross-sectional)	Patients enrolled in the FibroQoL study, a psychoeducational program for fibromyalgia N: 157 Sex: 98.1% female	18–75	DSM-IV IPDE Screener (self-report)	Identified patients with fibromyalgia according to ARC criteria from a database at the Viladecans Hospital	Patient-reported outcome • Functional status according to the FIQ	• 65% patients with fibromyalgia had a possible PD • Compared to without, patients with fibromyalgia and comorbid probable PD had higher FIQ scores (59.2 vs. 51.1, *p* < 0.001)
Uguz et al. (65) Turkey (Case-control)	Patients attending a Rheumatology Outpatient Clinic of the Research and Training Hospital of Necmettin Erbakan University N: 30 (with PD) N: 112 (without PD) N: 60 (controls) Sex: 93.1% female	42.64 (10.64)	DSM-III-R SCID-II (expert)	Fibromyalgia according to ARC criteria (expert diagnosis)	Patient-reported outcome • QoL according to the WHO QoL Assessment-Brief	• Patients with fibromyalgia and comorbid PD had lower physical health subscale scores [M = 44.90 (SD = 16.47) compared to patients with no PD [M = 51.57 (SD = 18.66) and controls [M = 77.65 (SD = 11.51, *p* < 0.001) • Patients with fibromyalgia and comorbid PD had lower psychological health subscale scores [M = 45.43 (SD = 20.32) compared to patients with no PD [M = 59.84 (SD = 16.26) and controls [M = 72.16 (SD = 13.48, *p* < 0.001) • Patients with fibromyalgia and comorbid PD had lower social relationship subscale scores [M = 42.40 (SD = 14.85) compared to patients with no PD [M = 57.96 (SD = 17.58) and controls [M = 71.48 (SD = 15.31, *p* < 0.001) • No statistically significant differences between groups on subscale scores for environment
**Clinician-reported outcomes**						
Dersh et al. (58) USA (Prospective cohort)	Patients before and after receiving treatment in the PRIDE functional restoration program N: 1,323 Sex: 38.3% female	41.9 (9.6)	DSM-IV SCID-II (expert)	Grouped musculoskeletal/spinal disorders according to pain/injury site: cervical and/or thoracic, lumbar, multiple spinal, multiple musculoskeletal with at least one spinal (expert diagnosis)	Clinician-reported outcome • Treatment non-completion for MSDs	• Patients with MSDS with comorbid antisocial [OR = 2.4 (95%CI, 1.2–4.8), *p* = 0.011] and dependent PDs [OR = 2.3 (95%CI, 1.3–4.1), *p* = 0.004] were more likely to be program *non-completers* than patients without these PDs
Howard et al. (52) USA (Prospective cohort)	Patients before and after receiving treatment in the PRIDE functional restoration program N: 3,052 (total) N: 2,367 (completer) N: 685 non-completer group M: 46.3% female (completer) M: 46.4% female (non-completer)	Completer: 45.1 (9.62) Non-completer: 45.2 (10.48)	DSM-IV SCID-II (expert)	Musculoskeletal/spinal disorders according to pain/injury sites: cervical, thoracic/lumbar, multiple spinal, multiple musculoskeletal, upper extremity, lower extremity upper and lower but no spine (expert diagnosis)	Clinician-reported outcome • Treatment non-completion for MSDs	• Compared to completers, patients with MSDs and comorbid Cluster B PD had higher odds of treatment non-completion [OR = 1.62 (95%CI, 1.22-2.14), *p* < 0.001] • No significant associations between Clusters A or C PDs and treatment completion status
Perish (81) USA (Prospective cohort)	Patients before and after receiving treatment in the ALBP at The University of Texas Southwestern Medical Center N: 53 (total) N: 30 (completer) N: 23 (non-completer) Sex: 49.1% female	41.58 (11.19); 19 to 63	DSM-IV SCID-II (expert)	Acute low back pain (expert diagnosis)	Clinician-reported outcome • Treatment non-completion for MSDs	• 51% of patients had PD • No significant associations between PD and treatment completion status among patients with acute low back pain
Frankenburg and Zanarini (87) USA (Prospective cohort)	Patients enrolled in the MSAD study N: 264 N: 200 (ever remitted) N: 64 (never remitted) Sex: 80.0% female (ever remitted) Sex: 82.8% female (never remitted)	Ever remitted: 32.5 (5.8) Never remitted: 34.5 (5.8)	DSM-III-R DIB-R (expert)	Osteoarthritis and chronic back pain (expert diagnosis)	Clinician-reported outcome • Borderline PD remission status on MSD outcomes after 6-years of follow-up	• Compared to patients with borderline PD who remitted, patients who never remitted were more likely to have chronic back pain [RRR = 1.68 (95%CI, 1.25-2.10), *p* < 0.001] and osteoarthritis [RRR = 2.29 (95%CI, 1.11-4.73), *p* = 0.25] Age/sex/race did not significantly contribute to the models
Keuroghlian et al. (42) USA (Prospective cohort)	Patients enrolled in the MSAD study N:264 N: 134 (ever recovered) N: 97 (never recovered) Sex: 80.7% female	33.0 (SD = 5.9)	DSM-III-R DIB-R (expert)	Osteoarthritis and chronic back pain (expert diagnosis)	Clinician-reported outcome • Borderline PD remission status on long-term MSD outcomes after 16 years of follow-up	• By the 16-year follow-up, the comorbidity of PD and osteoarthritis among never recovered and ever recovered (15.5% vs. 4.0% vs. at study baseline) increased to approximately 11.9% and 26.8%, respectively (*p* < 0.0063) • By the 16-year follow-up, the comorbidity of PD and chronic back pain among never recovered and ever recovered (45.7% vs. 39.2% at study baseline) increased to approximately 57.7% vs. 47.8%%, respectively (*p* < 0.0063)
**Work-related outcomes**						
Dersh et al. (58) USA (Prospective cohort)	Patients before and after receiving treatment in the PRIDE functional restoration program N: 1,323 Sex: 38.3% female	41.9 (9.6)	DSM-IV SCID-II (expert)	Grouped musculoskeletal/spinal disorders according to pain/injury site: cervical and/or thoracic, lumbar, multiple spinal, multiple musculoskeletal with at least one spinal (expert diagnosis)	Work-related outcomes • Work status at one-year follow-up	• Patients with MSDs with comorbid paranoid PD were less likely to have returned to work [OR = 1.6 (95%CI, 1.1–2.3), *p* = 0.011] or retained work [OR = 1.6 (95%CI, 1.1–2.2), *p* = 0.011], after one-year of follow-up
Gatchel et al. (48) USA (Prospective cohort)	Patients before and after receiving treatment in the PRIDE functional restoration program N: 152 N: 129 (return-to-work) N: 23 (no return-to-work) F: 35% female (return-to-work) F: 43% female (no-return-to-work)	Return-to-work: 35.7 (8.9) No return-to-work: 37.1 (7.2)	DSM-IV SCID-II (expert)	Chronic low back pain including degenerative disk disease, lumbar radicular syndrome, postoperative epidural fibrosis, segmental instability, and non-specific back pain (expert diagnosis)	Work-related outcomes • Return-to-work status at one-year follow-up	• 58% of patients with MSDs had PD • PD was not significantly associated with return-to-work status among patients with MSDs
**Hospital admissions**						
Fok et al. (94) UK (Retrospective case-control)	Patients receiving care from the SLaM service N: 7,677 Sex: 55.75% female	36.32 (14.69)	ICD-10 PD Diagnoses searched using CRIS at SLaM and GATE language processing software from case notes/correspondence	ICD-10 general hospital admission/discharge diagnoses using linked HES data	Hospital admission • Hospital admissions for MSD-related causes	• Patients with PD had more hospital admissions for any ICD-10 MSD compared to the standard population [SAR = 2.98 (95% CI 2.72–3.26), *p* < 0.05] during the observation period. • The admissions for women were slightly elevated among women with PD [SAR = 3.25 (95%CI, 2.88–3.65), *p* < 0.05]) than men with PD [SAR = 2.67 (95%CI, 2.31–3.07), *p* < 0.05] • SARs were age-sex adjusted; standard population were age-sex-fiscal year adjusted
Schubert et al. (79) USA (Cross-sectional)	Consecutive admissions to a psychiatry ward at Metro Health Medical Center, Cleveland, Ohio N: 532 (total) N: 222 (psychiatric dx without physical dx: N: 310 (psychiatric dx + physical dx) Sex: 66% female	Total: mean age range 30-46 Psychiatric dx no physical dx: 33.2 (10.5) Psychiatric dx + physical dx:43.0 (15.3)	ICD-9 Psychiatrist diagnosis	Diagnoses of musculosystem and connective tissue diseases ascertained from hospital records according to ICD-9	Hospital admission • Length of hospital stay in hospital	• 6.6% of patients who were admitted to hospital for an MSD had PD • No significant association between PD and length of stay hospital
**Financial costs**						
Gumà-Uriel et al. (69) Spain (Cross-sectional)	Patients enrolled in the FibroQoL study, a psychoeducational program for fibromyalgia N: 157 Sex: 98.1% female	18–75	DSM-IV IPDE Screener (self-report)	Identified patients with fibromyalgia according to ARC criteria from a database at the Viladecans Hospital	Financial costs • Direct healthcare utilization costs	• 65% patients with fibromyalgia had a possible PD • Compared to without, people with fibromyalgia and possible PD had higher direct costs including primary care services, and specialist services (all *p* < 0.05) • No significant associations between PD and indirect costs among patients with fibromyalgia
**Other**						
Ericsson et al. (71) Sweden (Prospective cohort)	Chronic pain patients attending a National Social Insurance Hospital N: 184 Sex: 72.8% female	43.4 (10.8)	DSM-III-R SCID-II Screen (self-report)	Grouped chronic pain at multiple musculoskeletal sites/localized neck/back/extremity pain identified from a review of insurance records	Other disability indicator • Disability status according to disability insurance • record reviews after two-and-a-half years’ following index examination	• Possible PD not significantly associated with disability status among patients with MSDs at follow-up
Gatchel et al. (49) USA (Prospective cohort)	Patients entering the PRIDE functional restoration program N: 1,489 Sex: 42.8% female	42.3 (9.7)	DSM-IV SCID-II (expert)	Grouped musculoskeletal/spinal disorders (expert diagnosis)	Other disability indicator • “Disability profile derived from the MMPI	• Compared to without, patients with MSDs and a MMPI “disability profile” were more likely to have comorbid PD [OR = 4.7 (95%CI, 2.8–7.7, *p* = nr)

ALBP, acute low back pain program; CI, confidence interval; CRIS, clinical record interactive search; DIB-R, diagnostic interview for borderlines-revised; DSM, diagnostic and statistical manual of mental disorders; Dx, diagnosis; FIQ, fibromyalgia impact questionnaire; GATE, generalized architecture for text engineering; HES, hospital episodes statistics; ICD, international classification of diseases and related health problems; IPDE, international personality disorder examination; MMPI, minnesota multiphasic personality inventory; MSAD, McLean Study of Adult Development; MSD, Musculoskeletal disorders; nr, not reported; NSAID, non-steroidal anti-inflammatory drug; OR, odd ratio; PD, personality disorder; POINT, pain and opioids IN treatment; PRIDE, productive rehabilitation institute of Dallas for Ergonomics; QoL, quality of life; RRR, relative risk ratio; SCID-II, structured clinical interview for DSM Axis II personality disorders; SLaM, South London and Maudsley NHS Foundation Trust; WHO, World Health Organization.

## 3. Results

The results of the study identification selection process are presented in Figure 1. For the database searching, the Medline Complete search yielded 1,483 records; CINAHL Complete and PsycInfo each yielded 535 and 1,176 records, respectively. After removing duplicates, 2,307 records were screened and 2,139 were excluded. There were 168 full-text articles were assessed for eligibility. Of those, 127 studies were excluded with reasons (see Figure 1), resulting in 41 eligible records from the database searching (*n* = 8 reviews; *n* = 33 individual studies including a thesis). Searching the references of included reviews (*n* = 8) yielded an additional 430 records; of those, 24 were assessed for eligibility, 15 were excluded with reasons, and 11 were identified as eligible (*n* = 2 reviews; *n* = 9 individual studies). One additional article by the current group of authors was also included. Finally, the Google search yielded 38 potentially relevant sources, of which 4 were eligible (*n* = 4 individual studies including a thesis). In total, 57 articles were included in this scoping review.

### 3.1. Study characteristics

We identified 57 individual studies that met the inclusion criteria. Briefly, these included 10 reviews and 47 individual studies/analyses; the latter included two published theses, which were considered sources of gray literature. No other forms of gray literature were identified. The characteristics of the individual studies are presented as Supplementary Table 3.

The majority (*n* = 29) of the 47 individual studies were conducted in the United States of America (USA) (11, 40, 42, 46–59). There were four studies deriving from Germany (41, 45, 60, 61), three studies each from Australia (44, 62, 63) and Turkey (64–66); two studies each were from Norway (67, 68), Spain (69, 70), and Sweden (71, 72), and one study each from Italy (73), and the UK (74).

There were 26 studies that employed cross-sectional designs (22, 32, 40, 41, 43–45, 47, 50, 53, 59–62, 64, 67, 68, 70, 72, 73, 75–79). Of those, six studies conducted analyses at the admission phase of an intervention (51, 55–57, 69, 80). In addition, 11 were prospective cohort studies (11, 42, 46, 48, 49, 51, 52, 58, 71, 81, 82), of which, six conducted outcome analyses in cohorts of patients with MSDs (48, 49, 51, 52, 58, 81). Two further cohort studies were retrospective (54, 74), and there were seven case-control studies (64–68, 70).

To ascertain PDs, the Structured Clinical Interview for DSM Axis II Personality Disorders (SCID-II) was the most commonly used semi-structured interview with most stating it was administered by either mental health professionals (48, 51, 55–58, 61, 64, 66, 81) or trained interviewers (44, 63, 80). Other methods to identify PD included the interrogation of medical records or chart reviews according to ICD-9 or ICD-10 criteria (54, 74, 79), and clinical impressions (according to DSM criteria) based on collateral sources such as psychological interviews and testing and/or flowcharts (59, 75). In terms of self-reported assessments, the Millon Clinical Multiaxial Inventory (MCMI) was used in one study (70), and one further study used a non-validated questionnaire based on traits from diagnostic criteria for obsessive-compulsive PD (78). Finally, a number of studies selected specific items from, or used the entire Iowa Personality Disorder Screen (67, 68), International Personality Disorder Examination (IPDE) (53, 62, 69, 77), or the Personality Diagnostic Questionnaire-4 (PDQ-4) (22, 47), or the SCID-II Screen (questionnaire only) (71, 72).

For the identification of MSDs, in clinical settings, diagnoses were mostly performed by experts such as physicians, specialists, or multidisciplinary teams (11, 41, 42, 45–49, 51, 52, 55–57, 60, 61, 63, 65, 66, 70, 72, 73, 80), or identified from medical history records (69, 71, 74, 75, 79). In population-based settings, it was more common for MSDs to be self-reported (32, 40, 43, 44, 50, 67, 68, 76, 77, 82).

### 3.2. What is known regarding the comorbidity between PDs and MSDs?

We identified 10 existing reviews that reported on PDs and physical comorbidities (19, 21, 26–31, 83, 84). The majority of individual studies that were reviewed had observational designs from population-based (31), clinical (83–85), or a mixture of these settings (18, 19, 27–30). Associations between PD and MSDs, specifically, were reported to varying extents, depending on the focus of review. Yet, the reviews highlighted associations between PDs and MSDs such as chronic back pain (21, 27, 30), arthritis (19, 26, 28, 31), myalgia or fibromyalgia (83, 84), or bone mineral density (18). Of note, there were commonalities and overlap between these existing reviews. As highlighted by others, and given the similarities of existing reviews, there are opportunities to reduce duplication of research efforts in the future, by developing protocols for reviews and publishing them in via freely available platforms (33, 86). In addition—acknowledging that the field of evidence synthesis and review methodologies has advanced exponentially over the past decade (33, 86)—we identified inconsistencies in the completeness of reporting the approach for searching and selecting articles, as well as extracting, analyzing, and presenting results. There were no meta-analytic studies.

The results of relevant individual studies/analyses, including those identified from the reviews are synthesized in the following sections and presented in Table 1.

### 3.2.1. Conditions of the back

The comorbidity of “any” PD ranged between 43.6% and 69.6% among patients with back conditions in three clinically based cross-sectional studies (57, 75, 80). In addition, paranoid PD appeared to be the most common specific PD in two separate studies among patients with back conditions enrolled in the Productive Rehabilitation Institute of Dallas for Ergonomics (PRIDE) in the USA (57, 80). Furthermore, in one clinical study, the proportion of PDs among patients with low back pain was examined according to their smoking status. A higher proportion of smoking versus non-smoking patients had histrionic PD (61.7 versus 38.3%), a higher proportion of non-smoking patients versus smoking had obsessive-compulsive PD (77.2 versus 22.8%), and with no differences observed between smoking status and dependent PD (59).

Separately, only one study was detected that examined the comorbidity of back conditions in patients with PDs. In the clinical longitudinal study—the McLean Study of Adult Development (MSAD)—patients with borderline PD plus obesity had an increased risk of chronic back pain six-years after the index admission compared to patients without obesity (58.1 versus 39.0%) (11). While there is scant evidence examining back conditions in patients with PDs longitudinally, is it plausible that recovery from PDs may be hindered by physical morbidity or vice versa.

Four population-based cross-sectional studies were uncovered, which examined the comorbidity of PDs and back conditions—each with varying aims and approaches. In the National Comorbidity Survey Replication, 27.2% of people with back conditions had probable borderline/antisocial PDs (grouped using these items on IPDE screener) (53). Additional analyses showed people with back conditions had higher borderline PD symptomatology than those who reported no history, however the differences were not significant (77). Separately, in a population-based survey of people with chronic back pain, 15.5% had any PD, with Cluster C PDs being the most common (60).

### 3.2.2. Conditions of the joints

In brief, more studies were uncovered that examined the comorbidity of PDs and joint conditions, namely arthritis, in population-based settings than clinical settings.

The three clinical studies identified (11, 22, 73) all varied in terms of methodological approach, yielding various findings. In one of them, 87% of patients with diagnosed rheumatoid arthritis had a PD, 40% had obsessive-compulsive and borderline PDs each, and 7% each had schizoid and dependent PDs (73). In another study, probable PD was not significantly associated with self-reported rheumatoid arthritis in patients with opioid dependence (22). In the only clinically based longitudinal analysis, patients with borderline PD and comorbid obesity had an increased risk of osteoarthritis after 6-years of follow-up compared to patients without comorbid obesity (24.3% versus 4.2%) (11).

In the population-based setting, there was evidence of comorbidity between PDs and arthritis from seven cross-sectional studies (32, 40, 43, 44, 50, 76, 77), particularly for the “Cluster B” PDs—however in one study—the association was mediated by obesity (43). In the only longitudinal analysis (Waves I and II of the NESARC), PD did not significantly predict incident arthritis among people aged 55 + years with an anxiety disorder (82).

### 3.2.3. Soft tissue conditions

The comorbidity of PDs and soft tissue conditions (namely fibromyalgia/muscular pain) were examined most frequently in clinical settings including three cross-sectional studies and two case-control studies. In these studies, the frequency of “any” PD/probable PD, which likely varied in part due to methodological differences including assessment of PDs, ranged between 8.7 and 65.0% (47, 61, 64, 66, 69). Meanwhile, PDQ scores were not significantly associated with fibromyalgia among patients with opioid dependence (22).

In the population-based setting, studies varied considerably in terms of PDs of focus in relation to soft tissue conditions. One cross-sectional study reported that of people with fibromyalgia, 26.8% had possible obsessive-compulsive PD (78). In a separate case-control study, of people who screened positive for a PD, 33% reported muscular pain compared to 22% of control participants and 4% and 2% reported fibromyalgia, respectively (67). In separate analyses from the same cohort, 37% who screened positive for avoidant PD in particular (n = 280) reported muscular pain, compared to 20% of control participants (n = 1,400) who screened negative (68).

### 3.2.4. Disorders of bone density and structure

Evidence for the comorbidity between PDs and bone health is only emerging. Two separate cross-sectional studies from a clinical cohort of patients undergoing specialized treatment for borderline PD (41, 45), and one from a population-based (63) were identified. Data from these studies suggest that women with PDs have reduced bone mineral density—although it is not clear whether other comorbidities are driving these associations (41, 45, 63). Furthermore, osteoporosis was not more prevalent among women with than without PDs in the population-based study (63). There were no studies that examined PDs and BMD in populations other than women, or investigated associated fracture.

### 3.2.5. Other MSDs

Several additional clinical studies examined a range of, or heterogenous MSDs in relation to PDs, which were not described in the previous sections.

Two separate cross-sectional studies examined patients who entered the PRIDE program with heterogenous musculoskeletal conditions at various sites (51, 56). First, 70.0% of patients had a PD (56) with the three most frequent being paranoid PD (31.0%), borderline PD (27.5%), and histrionic PD (17.8%) (56). In a subsequent study (dissertation), the percentage of PDs did not appear to differ according to the musculoskeletal region involved in the condition (51)—suggesting PDs may be clinically meaningful diagnoses in patients, regardless of the specific musculoskeletal site. In a clinical cross-sectional study of patients with fibromyalgia (*n* = 92), myalgia (*n* = 44), spine/joint diagnoses (*n* = 111), and depression (*n* = 169)—all patient groups scored below diagnostic thresholds for PD (SCID-II) (72).

Elsewhere, in a case-control study of patients with osteoarthritis with central sensitization (CS), osteoarthritis without CS, fibromyalgia and control participants without these conditions, there was no clear differences between clinically significant MCMI profiles and the MSD groups (70).

### 3.3. What is known regarding the burden associated with PD and MSD comorbidity?

The identified studies that examined the burden associated with PDs and specific MSDs are synthesized into categories of outcome types in the following sections and in Table 2.

### 3.3.1. Morbidity

Three separate studies examined the role of PDs and MSDs comorbidity in relation to opioid medication use across clinical and population settings. One population-based cross-sectional study of people prescribed opioid medications for a range of MSDs (including arthritis, chronic back/neck pain, and fibromyalgia) found that people with probable borderline PD had higher use of oral morphine equivalent, daily benzodiazepines, and accidental overdose (62). Separately, evidence from a clinical retrospective cohort study showed that patients with MSDs (chronic back conditions) who were long-term users of opioids were more likely to have a PD than patients who used non-steroidal anti-inflammatory medications (54). In addition, in a clinical prospective cohort of patients with borderline PD, having a comorbid MSD (chronic back pain, fibromyalgia, and osteoarthritis) was predictive of opioid medication use (46).

### 3.3.2. Patient-reported outcomes

Few studies employed patient-reported outcome measures such as measures of symptomatology, functioning, and quality of life domains to examine burden associated with the comorbidity of PDs and MSDs.

A clinical, cross-sectional study from the PRIDE showed that patients with MSDs who reported the highest pain anxiety symptom scores (according to the Pain Anxiety Symptom Scale) also had the highest frequency of PDs in a dose-response type pattern (55). Elsewhere, results from a clinical, cross-sectional analysis showed that patients with fibromyalgia had poor functional impairment (as measured by the Fibromyalgia Impact Questionnaire) (69), while a separate clinical case-control study reported patients with fibromyalgia and a comorbid PD had poorer physical and psychological health and social relationships on the WHOQOL-BREF compared those without PDs (65).

### 3.3.3. Clinician-reported outcomes

Several studies were identified that examined clinician-reported outcome measures in relation to the comorbidity of PDs and MSDs such as the status of prescribed treatment completion for MSDs or the remission status of PDs.

Three clinical longitudinal studies examined PDs as predictors of treatment completion among patients entering prescribed programs for the treatment of MSDs (51, 58, 79). Two studies using data from the PRIDE reported a higher frequency of PDs among people who did not complete their prescribed treatment (51, 58). A third separate study, did not find any association between PDs and treatment completion status (81).

Elsewhere, two separate longitudinal analyses from a clinical prospective cohort (MSAD) revealed patients with non-remitted borderline PD had increased risk of MSDs over the long term (42, 87)—suggesting the severity and course of PDs may have adverse effects on musculoskeletal health over time.

### 3.3.4. Work-related outcomes

Of two longitudinal analyses from a clinical prospective cohort (PRIDE)—which examined PDs as predictors of work-related outcomes among patients—the first analyses showed no significant association between PDs and return-to-work status among patients with chronic low back pain (48), while the second, revealed patients with diverse MSDs and comorbid PDs were less likely to have returned to work, or retained work, by the one-year follow up (58).

### 3.3.5. Hospital admissions

Only two studies were detected that considered the role of PDs and MSDs in relation to hospital admissions. In a clinical retrospective case-control study, people with PDs had elevated hospital admissions for MSD-related causes compared to those without PDs (74). An earlier clinical study found that PDs did not appear to contributed to a lengthier hospital stay due to MSDs (79).

### 3.3.6. Financial costs

Only one study was uncovered that examined costs associated with PDs and MSDs. Specifically, one clinical cross-sectional analysis found that compared to patients without fibromyalgia, those with PDs plus fibromyalgia, had higher direct (i.e., primary care and specialist costs) but not indirect healthcare costs (65).

### 3.3.7. Other indicators

There were two additional studies that examined differing indicators of disability in relation to the research questions. In a clinical longitudinal study of chronic pain patients with MSDs, PDs did not appear to predict disability status according to insurance records (71). Separately, in PRIDE, patients with MSDs with a “disability profile” (derived from the MMPI) were more likely to have comorbid PD (49).

## 4. Discussion

In this scoping review, we examined the comorbidity between PDs and MSDs and their associated burden—scoping evidence from 10 reviews and 47 individual analyses. Whilst the findings vary due to methodological differences including sample size, study population, and assessment methods for PDs and MSDs—overall we found evidence of comorbidity between PDs and chronic back/neck/or spine conditions, arthritis, fibromyalgia, and reduced bone mineral density to varying extents. We also uncovered that there is only scant research that examines the potential burden associated with the comorbidity between PDs and MSDs from various outcome themes including morbidity/mortality, patient-reported outcomes, clinician-reported outcomes, work-related outcomes, hospital admissions, and financial costs. A discussion of the findings in relation to the two remaining research questions are presented in the following sections.

### 4.1. What are the knowledge gaps in relation to this topic? What recommendations for future research can be made?

Evidence from clinical cross-sectional studies (57, 75, 80) and one longitudinal study (11) suggest high levels of comorbidity between PDs and back conditions. However, it appears the evidence for associations between PDs and back conditions is both heterogeneous and lacking in the general population setting, suggesting further research in these settings is needed. Similarly, given the increasing population-based cross-sectional evidence for associations between PDs and arthritis, further longitudinal studies are now needed to ascertain causality and underlying mechanisms.

We also detected evidence that suggests potentially high occurrences of PDs among patient populations with fibromyalgia (47, 61, 64, 66, 69). There is a suggestion for specific associations between “Cluster C” PDs and fibromyalgia, but this evidence derives from limited cross-sectional studies (47, 65, 69) and a case-control study in the general population (68). People with comorbid PD and fibromyalgia also tended to report poorer functional status (69) and poorer quality of life (65). As such, further epidemiological studies using population-based samples might provide greater certainty in terms of the association, directionality, and outcomes of these two groups of disorders.

Separately, there is spare research on the associations of PDs and bone mineral density. In their brief report, Williams et al. highlighted that specific agents such as selective serotonin reuptake inhibitors, anticonvulsants, and antipsychotics are associated with low bone mass (88) and increased bone loss (89). In addition, they are commonly prescribed pharmacotherapy for PD (90). As such, further research is needed to determine if people with PDs may be susceptible to osteoporosis and fragility fractures, and to investigate possible mechanisms of which, is poorly understood. Thus, the relationship between PDs and bone health warrants further research attention, given the continuing prevalence and burden of osteoporosis and associated fragility fractures in the population.

More broadly, the longitudinal course of PD and MSD comorbidity is under explored, as are their underlying mechanisms. It is likely that PDs and MSDs have shared and non-shared risk (and protective) factors, however, they are poorly understood. To date, explanations linking PD and types of chronic pain more broadly (rather than MSDs *per se*) are consistent with stress-diathesis and biopsychosocial models (23, 91, 92). These models strongly consider the role of psychological and social factors and their interaction with biological factors in the etiology and maintenance of pain. Thus, a biopsychosocial approach offers a model to conceptualize and conduct further research on the associations between, and the course of, PD and MSDs over time ensuring that the interrelationships of physical, psychological, and social factors are considered. Also, future studies may further explore the potential role of CS—a process of the nervous system that is understood to be implicated in the development or maintenance of pain—in the comorbidity of PD and MSDs, which currently remains unclear (70). Also, specific explanatory factors in the relationship between PDs and MSDs that might warrant further exploration include lifestyle factors such as smoking and obesity status, along with the impact of the course/chronicity and severity of PDs on MSD trajectories and vice versa. Separately, this scoping review revealed that the burden associated with PDs and MSDs is poorly understood. Still, several studies showed that opioid medication use was common among people with comorbid PDs and MSDs (46, 54, 62). These studies identified the importance of balancing the risks of appropriate pain management for MSDs with the potential for overdose as a consequence of opioid use among potentially vulnerable individuals with PDs.

Elsewhere, work-related outcomes associated with PDs and MSDs remain unclear. Interestingly, in one study deriving from the SPAN, current employment status was associated with a weaker negative relationship between borderline PD features and self- and informant- ratings of subjective physical health (i.e., not MSDs specific)—suggesting being employed may mitigate the adverse impacts of borderline PD features on general physical health (93). The authors called for further longitudinal research to examine the course and moderators of the relationship between PDs and physical health in general, including the role of occupational functioning (93). As such, it is suggested that an improved understanding of the role of employment status, work environments, and occupational functioning is needed for the prevention or management of PDs and MSDs specifically.

There is only a paucity of research that utilizes patient reported outcome measures to ascertain the burden of PDs and MSDs. As such, further research is needed to examine experiences from the view of patients, which goes beyond measuring patient-reported outcomes in single classes of conditions/diseases. In addressing these gaps in the literature, utilizing appropriate and psychometrically sound instruments and analytic techniques may ensure that evidence produced on this topic is robust, of high quality, and responsive to identifying clinically important changes over time (where appropriate).

There is also scant literature investigating these comorbidities in relation to the impact on hospital admissions or utilization of other healthcare services and costs—further research on these outcomes may be beneficial for planning health service needs. Furthermore, to the authors knowledge, the is no existing evidence examining MSDs as an underlying cause of mortality among people with PDs or vice versa—this may be important research to undertake, given that previous research has shown premature mortality in individuals with PDs (94).

Finally, we propose that systematic reviews involving critical appraisal and meta-analyses are appropriate next steps to strengthen the evidence base on what is known in this field. However, it is acknowledged that the evidence to date, which derives from studies examining diverse populations with various methodological approaches, makes it challenging to conduct systematic reviews and meta-analyses, which are considered higher forms of evidence. Finally, given the extent of the published gray literature detected were dissertations, and there were no published documents uncovered from government agencies—this suggests improved awareness of these comorbidities in governmental and public health settings is needed.

Taken together, the existing evidence highlights a plausible need for the identification of psychological concerns in MSD treatment settings among people with PD. This may reduce the need for a patient to navigate multiple systems, which may in turn, reduce inappropriate referrals, frequent presentations in primary and emergency care, and enhance treatment engagement. For example, there is evidence that a multidisciplinary functional restoration approach based on the biopsychosocial model, is effective in restoring both physical and psychosocial functional capacity (95). As such, further research is needed to investigate the mechanisms of action and the appropriateness of alike programs and interventions for people with PDs and MSDs.

### 4.2. Strengths and limitations of included studies

In terms of strengths, there were many analyses that utilized prominent data sources. Many studies (48, 51, 52, 55–58, 80) utilized data collected from the PRIDE, an on-going clinical and research program launched in 1983. Four (11, 42, 46, 87) derived from MSAD—a multifaceted longitudinal study of young adults with borderline PD (96). Of the population-based observational studies, five (32, 40, 50, 76, 82) utilized data from the NESARC, a representative study of the US population (97). In addition, two studies (53, 77) utilized data from the Part II NCS-R, a representative community-based household survey of mental disorders and correlates in the USA. A further study utilized data from the SPAN (43), a community-based study designed to investigate the role and impact of PD on later life outcomes including health, biology, and social adjustment (98). Elsewhere, two separate analyses (67, 68) derived from the HUBRO, a community-based cohort of individuals from Olso, Norway that was initiated by the Norwegian Institute of Public Health (99). A further two population-based analyses (44, 63) derived from the GOS, a community-based cohort in Australia (100). Also in Australia, the Pain and Opioids in Treatment (POINT) (62), is a community-based cohort of individuals who were prescribed with strong opioids for types of chronic pain, and investigating associations between mental disorders, chronic pain-related conditions and their associated outcomes (101).

There are also limitations and considerations to note. First, there was considerable differences in sample sizes informing analyses on the comorbidity of PD and MSDs that varied from n = 15 (73) to n = 43,093 (50, 76) and approximately one-third of the studies examined samples where either all, or majority (> 60%) of the sample were women (11, 41, 42, 44–46, 51, 61, 63–65, 69–73, 78, 87). Second, there was variability in the methods to ascertain PD, such as using expert ratings of semi-structured interviews versus self-reporting/use of screening instruments, which arguably lead to differences in frequencies of PD across studies. In addition, there was variation in definitions of MSDs between studies, even within the broad categorical groupings identified, which were guided by the ICD-10.

### 4.3. Strengths and limitations of this review

In terms of the strengths of the conduct of this review, we undertook a synthesis of the existing literature to understand the extent of, and the types of evidence on the comorbidity of PD and MSDs and associated burden. It was conducted according to a published protocol (35), current methodological guidance (33), and adheres to the PRISMA-ScR (36). Consistent with the remit of a scoping review, we did not undertake critical appraisal of the included studies, which precludes drawing conclusions about the quality of, and confidence in the evidence at this stage. Instead, this scoping study provides a broad, yet comprehensive introduction to the topic including the extent and types of available evidence. Therefore, readers may be guided by this scoping review to develop refined research questions, which more appropriately lend themselves to the conduct of systematic reviews and meta-analyses.

In terms of limitations, it was necessary to define a study population, scope, and inclusion criteria for this review, which was guided by the existing classifications of PD. It is acknowledged that the ICD-11, which will be implemented as the official reporting system commencing January 2022 has significantly reformed the section on PD. Therefore, future studies may build on the current review by considering how the findings could be transferable to the ICD-11 or trait models (e.g., see Conversano et al. (102) for a review on the Big-Five model, Eysenck’s and Cloninger’s models of personality in fibromyalgia).

As the focus of this review was MSDs—conditions of the back, joints, and soft tissue, and of bone density and structure in relation to PD—studies investigating non-MSDs-related chronic pain such as cancer pain, chronic fatigue syndrome, headache, inflammatory bowel disease, migraine, temporomandibular joint dysfunction, and others, were out of the scope of this review. Thus, it is acknowledged that the existing chronic pain literature may offer further insights into associations between PD and MSDs beyond what was discussed in the current review. Finally, the authors understand that the ICD-11 will include a new separate diagnostic code for fibromyalgia under the section for chronic pain rather than MSDs.

## 5. Conclusion

The findings from this scoping review provide insights into the extent and types of evidence concerning the comorbidity between PDs and MSDs. We revealed that the burden associated with comorbid PDs and MSDs is poorly understood. This scoping review might prompt further research into these disorders, along with their associated burden, and underlying mechanisms.

## Data availability statement

The original contributions presented in this study are included in the article/Supplementary material, further inquiries can be directed to the corresponding author.

## Author contributions

SEQ, HK-H, and LJW conceived and designed the study. All authors provided input into the methodology, significantly contributed to the interpretation of the findings, drafting the article, and approved the final version to be published.

## References

[1] Heikkinen J Honkanen R Williams L Leung J Rauma P Quirk S Depressive disorders, anxiety disorders and subjective mental health in common musculoskeletal diseases: a review. *Maturitas.* (2019) 127:18–25. 10.1016/j.maturitas.2019.05.011 31351516

[2] Whiteford HA Degenhardt L Rehm J Baxter AJ Ferrari AJ Erskine HE Global burden of disease attributable to mental and substance use disorders: findings from the global burden of disease study 2010. *Lancet.* (2013) 382:1575–86. 10.1016/S0140-6736(13)61611-6 23993280

[3] GBD 2016 Disease and Injury Incidence and Prevalence Collaborators. Global, regional, and national incidence, prevalence, and years lived with disability for 328 diseases and injuries for 195 countries, 1990-2016: a systematic analysis for the global burden of disease study 2016. *Lancet.* (2017) 390:1211–59. 28919117 10.1016/S0140-6736(17)32154-2 PMC5605509

[4] IHME. *Global Burden of Disease Results Tool.* Seattle, WA: Institute for Health Metrics and Evaluation (2020).

[5] Volkert J Gablonski T Rabung S. Prevalence of personality disorders in the general adult population in Western countries: systematic review and meta-analysis. *Br J Psychiatry.* (2018) 213:709–15. 10.1192/bjp.2018.202 30261937

[6] Winsper C Bilgin A Thompson A Marwaha S Chanen AM Singh SP The prevalence of personality disorders in the community: a global systematic review and meta-analysis. *Br J Psychiatry.* (2020) 216:69–78. 10.1192/bjp.2019.166 31298170

[7] Chanen A Thompson K. *The Age of Onset of Personality Disorders. In: Age of Onset of Mental Disorders: Etiopathogenetic and Treatment Implications.* New York, NY: Springer International Publishing (2018). p. 183–201. 10.1007/978-3-319-72619-9_10

[8] Kramer U Temes CM Magni LR Fitzmaurice GM Aguirre BA Goodman M Psychosocial functioning in adolescents with and without borderline personality disorder. *Personal Ment Health.* (2017) 11:164–70. 10.1002/pmh.1377 28597585

[9] Chanen A Jovev M Jackson H. Adaptive functioning and psychiatric symptoms in adolescents with borderline personality disorder. *J Clin Psychiatry.* (2007) 68:297–306. 10.4088/JCP.v68n0217 17335330

[10] Frankenburg F Zanarini M. Relationship between cumulative BMI and symptomatic, psychosocial, and medical outcomes in patients with borderline personality disorder. *J Pers Disord* (2011) 25:421–31. 10.1521/pedi.2011.25.4.421 21838559 PMC3203730

[11] Frankenburg F Zanarini M. Obesity and obesity-related illnesses in borderline patients. *J Pers Disord.* (2006) 20:71–80. 10.1521/pedi.2006.20.1.71 16563080

[12] Maclean J Xu H French M Ettner S. Personality disorders and body weight. *Econ Hum Biol.* (2014) 12:153–71. 10.1016/j.ehb.2013.10.002 24268441

[13] Byrne S Cherniack M Petry N. Antisocial personality disorder is associated with receipt of physical disability benefits in substance abuse treatment patients. *Drug Alcohol Depend.* (2013) 132:373–7. 10.1016/j.drugalcdep.2013.01.004 23394688 PMC3665619

[14] Wall K Kerr S Sharp C. Barriers to care for adolescents with borderline personality disorder. *Curr Opin Psychol.* (2021) 37:54–60. 10.1016/j.copsyc.2020.07.028 32853877

[15] Sanatinia R Middleton S Lin T Dale O Crawford M. Quality of physical health care among patients with personality disorder. *Personal Ment Health.* (2015) 9:319–29. 10.1002/pmh.1303 26248879

[16] World Health Organization. *Musculoskeletal Conditions.* Geneva: World Health Organization (2021).

[17] Williams A Kamper SJ Wiggers JH O'Brien KM Lee H Wolfenden L Musculoskeletal conditions may increase the risk of chronic disease: a systematic review and meta-analysis of cohort studies. *BMC Med.* (2018) 16:167. 10.1186/s12916-018-1151-2 30249247 PMC6154805

[18] Dixon-Gordon KL Whalen DJ Layden BK Chapman AL. A systematic review of personality disorders and health outcomes. *Can Psychol Can.* (2015) 56:168–90. 10.1037/cap0000024 PMC4597592 26456998

[19] Dixon-Gordon K Conkey L Whalen D. Recent advances in understanding physical health problems in personality disorders. *Curr Opin Psychol.* (2018) 21:1–5. 10.1016/j.copsyc.2017.08.036 28915400 PMC5847410

[20] Sansone R Sinclair J Wiederman M. Disability and borderline personality disorder in chronic pain patients. *Pain Res Manag.* (2010) 15:369–70. 10.1155/2010/952816 21165370 PMC3008661

[21] Sansone R Sansone L. Chronic pain syndromes and borderline personality. *Innov Clin Neurosci.* (2012) 9:10–4. PMC3280073 22347686

[22] Sansone R Whitecar P Wiederman M. Psychophysiological disorders among buprenorphine patients. *Int J Psychiatry Clin Pract.* (2009) 13:338–40. 10.3109/13651500903094575 24916946

[23] Turk D Monarch E. *Biopsychosocial Perspective on Chronic Pain.* 3rd Editio ed. New York, NY: Guilford Publications (2018). 10.1176/jnp.9.4.623

[24] Sansone R Sansone L. Borderline personality and the pain paradox. *Psychiatry.* (2007) 4:40–6. PMC2921236 20711327

[25] Gatchel R. Comorbidity of chronic pain and mental health disorders: the biopsychosocial perspective. *Am Psychol.* (2004) 59:795–805. 10.1037/0003-066X.59.8.795 15554853

[26] Dixon-Gordon K DWhalen D Layden B Chapman AL. A Systematic review of personality disorders and health Outcomes. *Can Psychol.* (2015) 56:188–90. 10.1037/cap0000024 PMC4597592 26456998

[27] Doering S. Borderline Personality disorder in patients with medical illness: a review of assessment, prevalence, and treatment options. *Psychosom Med.* (2019) 81:584–94. 10.1097/PSY.0000000000000724 31232916

[28] Dokucu M Cloninger C. Personality disorders and physical comorbidities. *Curr Opin Psychiatry.* (2019) 32:435–41. 10.1097/YCO.0000000000000536 31219842

[29] Douzenis A Tsopelas C Tzeferakos G. Medical comorbidity of cluster B personality disorders. *Curr Opin Psychiatry.* (2012) 25:398–404. 22744403 10.1097/YCO.0b013e3283558491

[30] Frankenburg F Zanarini M. Personality disorders and medical comorbidity. *Curr Opin Psychiatry.* (2006) 19:428–31. 10.1097/01.yco.0000228766.33356.44 16721176

[31] Quirk SE Berk M Chanen AM Koivumaa-Honkanen H Brennan-Olsen SL Pasco JA Population prevalence of personality disorder and associations with physical health comorbidities and health care service utilization: a review. *Personal Disord Theory Res Treat.* (2016) 7:136–46. 10.1037/per0000148 26461047

[32] Quirk SE El-Gabalawy R Brennan SL Bolton JM Sareen J Berk M Personality disorders and physical comorbidities in adults from the United States: data from the national epidemiologic survey on alcohol and related conditions. *Soc Psychiatry Psychiatr Epidemiol.* (2015) 50:807–20. 10.1007/s00127-014-0974-1 25314916

[33] Peters MD Marnie C Tricco AC Pollock D Munn Z Alexander L Updated methodological guidance for the conduct of scoping reviews. *JBI Evid Synth.* (2020) 18:2119–26. 10.11124/JBIES-20-00167 33038124

[34] Arksey H O’Malley L. Scoping studies: towards a methodological framework. *Int J Soc Res Methodol Theory Pract.* (2005) 8:19–32. 10.1080/1364557032000119616

[35] Quirk S Koivumaa-Honkanen H Honkanen R Heikkinen J Kavanagh B Williams L. Exploring the comorbidity of musculoskeletal and personality disorders among adults: a scoping review protocol. *Syst Rev.* (2021) 10:182. 10.1186/s13643-021-01721-6 34148544 PMC8215781

[36] Tricco AC Lillie E Zarin W O'Brien KK Colquhoun H Levac D PRISMA extension for scoping reviews (PRISMA-ScR): checklist and explanation. *Ann Intern Med.* (2018) 169:467–73. 10.7326/M18-0850 30178033

[37] Peters M Godfrey C McInerney P Munn Z Tricco A Khalil H. Chapter 11: scoping reviews (2020 version). In: Aromataris E, Munn Z editors. *JBI Manual for Evidence Synthesis*. JBI (2020). 10.46658/JBIMES-20-12

[38] World Health Organization. *The ICD-10 Classification of Mental and Behavioural Disorders: Clinical Descriptions and Diagnostic Guidelines.* Geneva: World Health Organization (1992).

[39] McGowan J Sampson M Salzwedel D Cogo E Foerster V Lefebvre C. PRESS peer review of electronic search strategies: 2015 guideline statement. *J Clin Epidemiol.* (2016) 75:40–6. 10.1016/j.jclinepi.2016.01.021 27005575

[40] El-Gabalawy R Katz L Sareen J. Comorbidity and associated severity of borderline personality disorder and physical health conditions in a nationally representative sample. *Psychosom Med.* (2010) 72:641–7. 10.1097/PSY.0b013e3181e10c7b 20508177

[41] Kahl KG Greggersen W Rudolf S Stoeckelhuber BM Bergmann-Koester CU Dibbelt L Bone mineral density, bone turnover, and osteoprotegerin in depressed women with and without borderline personality disorder. *Psychosom Med.* (2006) 68:669–74. 10.1097/01.psy.0000237858.76880.3d 17012519

[42] Keuroghlian A Frankenburg F Zanarini M. The relationship of chronic medical illnesses, poor health-related lifestyle choices, and health care utilization to recovery status in borderline patients over a decade of prospective follow-up. *J Psychiatr Res.* (2013) 47:1499–506. 10.1016/j.jpsychires.2013.06.012 23856083 PMC3884821

[43] Powers A Oltmanns T. Borderline personality pathology and chronic health problems in later adulthood: the mediating role of obesity. *Personal Disord Theory Res Treat.* (2013) 4:152–9. 10.1037/a0028709 22686464 PMC3443520

[44] Quirk SE Stuart AL Brennan-Olsen SL Pasco JA Berk M Chanen AM Physical health comorbidities in women with personality disorder: data from the geelong osteoporosis study. *Eur Psychiatry.* (2016) 34:29–35. 10.1016/j.eurpsy.2015.12.007 26928343

[45] Kahl KG Rudolf S Stoeckelhuber BM Dibbelt L Gehl H Markhof K Bone mineral density, markers of bone turnover, and cytokines in young women with borderline personality disorder with and without comorbid major depressive disorder. *Am J Psychiatry.* (2005) 162:168–74. 10.1176/appi.ajp.162.1.168 15625216

[46] Frankenburg F Fitzmaurice G Zanarini M. The use of prescription opioid medication by patients with borderline personality disorder and axis II comparison subjects. *J Clin Psychiatry.* (2014) 75:357–61. 24500123 10.4088/JCP.13m08557 PMC4019694

[47] Fu T Gamble H Siddiqui U. Psychiatric and personality disorder survey of patients with fibromyalgia. *Ann Depress Anxiety.* (2015) 2:7–9.

[48] Gatchel R Polatin P Mayer T Garcy P. Psychopathology and the rehabilitation of patients with chronic low back pain disability. *Arch Phys Med Rehabil.* (1994) 75:666–70. 10.1016/0003-9993(94)90191-0 8002766

[49] Gatchel R Mayer T Eddington A. MMPI disability profile: the least known, most useful screen for psychopathology in chronic occupational spinal disorders. *Spine.* (2006) 31:2973–8. 10.1097/01.brs.0000247807.10305.5d 17139230

[50] Goldstein RB Dawson DA Chou SP Ruan WJ Saha TD Pickering RP Antisocial behavioral syndromes and past-year physical health among adults in the United States: results from the national epidemiologic survey on alcohol and related conditions. *J Clin Psychiatry.* (2008) 69:368–80. 10.4088/JCP.v69n0305 18348594 PMC2958062

[51] Howard K. *Prevalence, Risk Factors and Treatment Outcomes for Various Musculoskeletal Injury Sites Associated With the Development of Chronic Occupational Disability.* Arlington, TX: The University of Texas at Arlington (2010).

[52] Howard K Mayer T Theodore B Gatchel R. Patients with chronic disabling occupational musculoskeletal disorder failing to complete functional restoration: analysis of treatment-resistant personality characteristics. *Arch Phys Med Rehabil.* (2009) 90:778–85. 10.1016/j.apmr.2008.11.009 19406297

[53] Braden J Sullivan M. Suicidal thoughts and behavior among adults with self-reported pain conditions in the national comorbidity survey replication. *J pain.* (2008) 9:1106–15. 10.1016/j.jpain.2008.06.004 19038772 PMC2614911

[54] Breckenridge J Clark J. Patient characteristics associated with opioid versus nonsteroidal anti-inflammatory drug management of chronic low back pain. *J Pain.* (2003) 4:344–50. 10.1016/S1526-5900(03)00638-2 14622692

[55] Brede E Mayer T Neblett R Williams M Gatchel R. The pain anxiety symptoms scale fails to discriminate pain or anxiety in a chronic disabling occupational musculoskeletal disorder population. *Pain Pract.* (2011) 11:430–8. 10.1111/j.1533-2500.2011.00448.x 21435161

[56] Dersh J Gatchel R Polatin P Mayer T. Prevalence of psychiatric disorders in patients with chronic work-related musculoskeletal pain disability. *J Occup Environ Med.* (2002) 44:459–68. 10.1097/00043764-200205000-00014 12024691

[57] Dersh J Gatchel R Mayer T Polatin P Temple O. Prevalence of psychiatric disorders in patients with chronic disabling occupational spinal disorders. *Spine.* (2006) 31:1156–62. 10.1097/01.brs.0000216441.83135.6f 16648753

[58] Dersh J Mayer T Gatchel R Towns B Theodore B Polatin P. Psychiatric comorbidity in chronic disabling occupational spinal disorders has minimal impact on functional restoration socioeconomic outcomes. *Spine.* (2007) 32:1917–25. 10.1097/BRS.0b013e31811329ac 17762302

[59] Fishbain D Lewis J Cole B Cutler R Rosomoff H Rosomoff R. Variables associated with current smoking status in chronic pain patients. *Pain Med* (2007) 8:301–11. 10.1111/j.1526-4637.2007.00317.x 17610452

[60] Gerhardt A Hartmann M Schuller-Roma B Blumenstiel K Bieber C Eich W The prevalence and type of axis-I and axis-II mental disorders in subjects with non-specific chronic back pain: results from a population-based study. *Pain Med.* (2011) 12:1231–40. 10.1111/j.1526-4637.2011.01190.x 21810166

[61] Thieme K Turk D Flor H. Comorbid depression and anxiety in fibromyalgia syndrome: relationship to somatic and psychosocial variables. *Psychosom Med.* (2004) 66:837–44. 10.1097/01.psy.0000146329.63158.40 15564347

[62] Campbell G Bruno R Darke S Degenhardt L. Associations of borderline personality with pain, problems with medications and suicidality in a community sample of chronic non-cancer pain patients prescribed opioids for pain. *Gen Hosp Psychiatry.* (2015) 37:434–40. 10.1016/j.genhosppsych.2015.05.004 26112358

[63] Williams L Quirk S Koivumaa-Honkanen H Honkanen R Pasco J Stuart A Personality disorder and physical health comorbidities: a link with bone health? *Front Psychiatry.* (2020) 11:602342. 10.3389/fpsyt.2020.602342 33363487 PMC7752862

[64] Kayhan F Küçük A Satan Y Ýlgün E Arslan Ş Ýlik F. Sexual dysfunction, mood, anxiety, and personality disorders in female patients with fibromyalgia. *Neuropsychiatr Dis Treat.* (2016) 12:349–55. 10.2147/NDT.S99160 26937190 PMC4762461

[65] Uguz F Kucuk A Cicek E Kayhan F Salli A Guncu H Quality of life in rheumatological patients: the impact of personality disorders. *Int J Psychiatry Med.* (2015) 49:199–207. 10.1177/0091217415582183 25930734

[66] Uguz F Ciçek E Salli A Karahan AY Albayrak I Kaya N Axis I and axis II psychiatric disorders in patients with fibromyalgia. *Gen Hosp Psychiatry.* (2010) 32:105–7. 10.1016/j.genhosppsych.2009.07.002 20114137

[67] Olssøn I Dahl A. Personality problems are considerably associated with somatic morbidity and health care utilisation. *Eur Psychiatry.* (2009) 24:442–9. 10.1016/j.eurpsy.2009.05.004 19540726

[68] Olsson I Dahl A. Avoidant personality problems – their association with somatic and mental health, lifestyle, and social network. A community-based study. *Compr Psychiatry.* (2012) 53:813–21. 10.1016/j.comppsych.2011.10.007 22146705

[69] Gumà-Uriel L Peñarrubia-María MT Cerdà-Lafont M Cunillera-Puertolas O Almeda-Ortega J Fernández-Vergel R Impact of IPDE-SQ personality disorders on the healthcare and societal costs of fibromyalgia patients: a cross-sectional study. *BMC Fam Pract.* (2016) 17:61. 10.1186/s12875-016-0464-5 27245582 PMC4888611

[70] López-Ruiz M Losilla JM Monfort J Portell M Gutiérrez T Poca V Central sensitization in knee osteoarthritis and fibromyalgia: beyond depression and anxiety. *PLoS One.* (2019) 14:e0225836. 10.1371/journal.pone.0225836 31805099 PMC6894784

[71] Ericsson M II Poston W Linder J Taylor J Haddock C Foreyt J. Depression predicts disability in long-term chronic pain patients. *Disabil Rehabil.* (2002) 24:334–40. 10.1080/09638280110096241 12017467

[72] Linder J Schüldt Ekholm K Lundh G Ekholm J. Long-term sick-leavers with fibromyalgia: comparing their multidisciplinarily assessed characteristics with those of others with chronic pain conditions and depression. *J Multidiscip Healthc.* (2009) 2:23–37. 10.2147/JMDH.S4659 21197344 PMC3004556

[73] Marcenaro M Prete C Badini A Sulli A Magi E Cutolo M. Rheumatoid arthritis, personality, stress response style, and coping with illness. A preliminary survey. *Ann N Y Acad Sci.* (1999) 876:419–25. 10.1111/j.1749-6632.1999.tb07666.x 10415637

[74] Fok M Chang C Broadbent M Stewart R Moran P. General hospital admission rates in people diagnosed with personality disorder. *Acta Psychiatr Scand.* (2019) 139:248–55. 10.1111/acps.13004 30689214

[75] Long D Filtzer D BenDebba M Hendler N. Clinical features of the failed-back syndrome. *J Neurosurg.* (1988) 69:61–71. 10.3171/jns.1988.69.1.0061 2967891

[76] McWilliams L Clara I Murphy P Cox B Sareen J. Associations between arthritis and a broad range of psychiatric disorders: findings from a nationally representative sample. *J Pain.* (2008) 9:37–44. 10.1016/j.jpain.2007.08.002 17890160

[77] McWilliams L Higgins K. Associations between pain conditions and borderline personality disorder symptoms: findings from the national comorbidity survey replication. *Clin J Pain.* (2013) 29:527–32. 10.1097/AJP.0b013e31826ab5d0 23328328

[78] Russek L Gardner S Maguire K Stevens C Brown EZ Jayawardana V A cross-sectional survey assessing sources of movement-related fear among people with fibromyalgia syndrome. *Clin Rheumatol.* (2015) 34:1109–19. 10.1007/s10067-014-2494-5 24481649

[79] Schubert D Yokley J Sloan D Gottesman H. Impact of the interaction of depression and physical illness on a psychiatric unit’s length of stay. *Gen Hosp Psychiatry.* (1995) 17:326–34. 10.1016/0163-8343(95)00065-Y 8522147

[80] Polatin P Kinney R Gatchel R Lillo E Mayer T. Psychiatric illness and chronic low-back pain. The mind and the spine–which goes first? *Spine.* (1993) 18:66–71. 10.1097/00007632-199301000-00011 8434327

[81] Perish MM. *A Review of the Biopsychosocial Characteristics in an Acute Low Back Pain Population (Order No. 3485496)*. Ann Arbor, MI: Argosy University/Dallas (2011).

[82] El-Gabalawy R Mackenzie C Pietrzak R Sareen J. A longitudinal examination of anxiety disorders and physical health conditions in a nationally representative sample of U.S. older adults. *Exp Gerontol.* (2014) 60:46–56. 10.1016/j.exger.2014.09.012 25245888

[83] Attademo L Bernardini F. Prevalence of personality disorders in patients with fibromyalgia: a brief review. *Prim Health Care Res Dev.* (2018) 19:523–8. 10.1017/S1463423617000871 29268809 PMC6452930

[84] Fietta P Fietta P Manganelli P. Fibromyalgia and psychiatric disorders. *Acta Biomed.* (2007) 78:88–95. 17933276

[85] Sansone R Butler M Dakroub H Pole M. Borderline personality symptomatology and employment disability: a survey among outpatients in an internal medicine clinic. *Prim Care Companion J Clin Psychiatry.* (2006) 8:153–7. 10.4088/PCC.v08n0305 16912818 PMC1540395

[86] Munn Z Peters M Stern C Tufanaru C McArthur A Aromataris E. Systematic review or scoping review? Guidance for authors when choosing between a systematic or scoping review approach. *BMC Med Res Methodol.* (2018) 18:143. 10.1186/s12874-018-0611-x 30453902 PMC6245623

[87] Frankenburg F Zanarini M. The association between borderline personality disorder and chronic medical illnesses, poor health-related lifestyle choices, and costly forms of health care utilization. *J Clin Psychiatry.* (2004) 65:1660–5. 10.4088/JCP.v65n1211 15641871

[88] Fernandes B Hodge J Pasco J Berk M Williams L. Effects of depression and serotonergic antidepressants on bone: mechanisms and implications for the treatment of depression. *Drugs and Aging.* (2016) 33:21–5. 10.1007/s40266-015-0323-4 26547857

[89] Rauma P Honkanen R Williams L Tuppurainen M Kröger H Koivumaa-Honkanen H. Effects of antidepressants on postmenopausal bone loss — A 5-year longitudinal study from the OSTPRE cohort. *Bone.* (2016) 89:25–31. 10.1016/j.bone.2016.05.003 27179631

[90] Mazza M Marano G Janiri L. An update on pharmacotherapy for personality disorders. *Expert Opin Pharmacother.* (2016) 17:1977–9. 10.1080/14656566.2016.1220542 27487174

[91] Weisberg J. Personality and personality disorders in chronic pain. *Curr Rev Pain.* (2000) 4:60–70. 10.1007/s11916-000-0011-9 10998717

[92] Weisberg J Vittengl J Clark L Gatchel R Gorin A. *Personality and Pain: Summary and Future Perspectives. In: Personality Characteristics of Patients With Pain.* Washington, DC: American Psychological Association (2004). p. 259–82. 10.1037/10376-012

[93] Cruitt P Boudreaux M Jackson J Oltmanns T. Borderline personality pathology and physical health: the role of employment. *Personal Disord Theory Res Treat.* (2018) 9:73–80. 10.1037/per0000211 27657166 PMC5311027

[94] Fok M Stewart R Hayes R Moran P. Predictors of natural and unnatural mortality among patients with personality disorder: evidence from a large UK case register. *PLoS One.* (2014) 9:e100979. 10.1371/journal.pone.0100979 25000503 PMC4085063

[95] Gatchel R Mayer T. Evidence-informed management of chronic low back pain with functional restoration. *Spine J.* (2008) 8:65–9. 10.1016/j.spinee.2007.10.012 18164455 PMC3237293

[96] Zanarini M Frankenburg F Hennen J Reich D Silk K. The McLean study of adult development (MSAD): overview and implications of the first six years of prospective follow-up. *J Pers Disord.* (2005) 19:505–23. 10.1521/pedi.2005.19.5.505 16274279

[97] Hasin D Grant B. The national epidemiologic survey on alcohol and related conditions (NESARC) waves 1 and 2: review and summary of findings. *Soc Psychiatry Psychiatr Epidemiol.* (2015) 50:1609–40. 10.1007/s00127-015-1088-0 26210739 PMC4618096

[98] Oltmanns T Rodrigues M Weinstein Y Gleason M. Prevalence of personality disorders at midlife in a community sample: disorders and symptoms reflected in interview, self, and informant reports. *J Psychopathol Behav Assess.* (2014) 36:177–88. 10.1007/s10862-013-9389-7 24954973 PMC4061059

[99] The Norwegian Institute of Public Health. *The Oslo Health Study (HUBRO).* (2019). Available online at: https://www.fhi.no/en/more/health-studies/landsomfattende-helseundersokelser-lhu/helseundersokelser/the-oslo-health-study-hubro/ (accessed January 18, 2021).

[100] Pasco J Nicholson G Kotowicz M. Cohort profile: geelong osteoporosis study. *Int J Epidemiol.* (2012) 41:1565–75. 10.1093/ije/dyr148 23283714

[101] Campbell G Mattick R Bruno R Larance B Nielsen S Cohen M Cohort protocol paper: the pain and opioids in treatment (POINT) study. *BMC Pharmacol Toxicol.* (2014) 15:17. 10.1186/2050-6511-15-17 24646721 PMC4000138

[102] Conversano C Marchi L Rebecca C Carmassi C Contena B Bazzichi LM Personality traits in fibromyalgia (FM): does FM personality exists? A systematic review. *Clin Pract Epidemiol Ment Heal.* (2018) 14:223–32. 10.2174/1745017901814010223 30294356 PMC6166394

