# Supplementary material for: Exploring the comorbidity between personality and musculoskeletal disorders among adults: A scoping review
Source: Front Psychiatry. 2023 Feb 2;13:1079106. doi: 10.3389/fpsyt.2022.1079106 (PMC9932280; doi:10.3389/fpsyt.2022.1079106)
Supplement: Supplementary file 1 [file Data_Sheet_1.docx]

| **General diagnostic requirements of personality disorder according to the Diagnostic and Statistical Manual of Mental Disorder (DSM-5)** (American Psychiatric Association, 2013)**:**  There are 10 specific types of PD that are organised into three Clusters. PDs are typically long-term patterns of behaviour and inner experiences that differ significantly from what is expected socially or culturally. The patterns of experiences/behaviours typically begin by late adolescence/early adulthood and cause significant distress/functioning. These include:  **Cluster A personality disorders**   - Paranoid PD - Schizoid PD - Schizotypal PD   **Cluster B personality disorders**   - Borderline PD - Histrionic PD - Narcissistic PD - Antisocial PD   **Cluster C personality disorders**   - Avoidant PD - Dependent PD - Obsessive-compulsive PD   **General diagnostic requirements for specific personality disorders according to the International Classification of Diseases (ICD-10)** (World Health Organization, 2021)**:**  Markedly disharmonious attitudes and behaviour, involving usually several areas of functioning, including affectivity, arousal, impulse control, ways of perceiving and thinking, and style of relating to others; these behavioural patterns are enduring, of long standing, and not limited to episodes of mental illness:   - Paranoid PD - Schizoid PD - Dissocial PD - Emotionally unstable PD   Impulsive type  Borderline type   - Histrionic PD - Anankastic PD - Anxious [avoidant] PD - Dependent PD - Other specific PD - PD, unspecified. |
| --- |

# **Supplementary Box 1:** *Definitions and criteria for personality disorder*

# **Supplementary Box 2:** *Common ICD-10 musculoskeletal diseases/conditions adapted from WHO musculoskeletal conditions (World Health Organization, 2021)*

| **Conditions of the back/spine (e.g. chronic back pain)**  Pain deriving from the spine, muscles, nerves or other structures in the back. It mostly arises as a direct result of disease or injury.  **Joint diseases (e.g. types of arthritis)**  A group of conditions involving inflammation of the joints, causing pain, stiffness, deformity/ disability. More than 100 different forms of arthritis are recognised, with common forms including osteoarthritis and rheumatoid arthritis.  **Soft tissue diseases (e.g. muscular pain/myalgia or fibromyalgia)**  Fibromyalgia is characterised by generalised aching, widespread tenderness/stiffness of joints, muscles or other tissues, and other somatic symptoms.  **Disorders of bone density and structure (e.g. osteopenia and osteoporosis)**  Disorders of bone density and structure occur when bone density thins/weakens. |
| --- |

# **Supplementary Table 1:** *Search strategy*

### **MEDLINE Complete via EBSCOhost platform**

| Search line | Index/keyword/combinations |
| --- | --- |
| S1 | (MH "Personality Disorders+") |
| S2 | (AB "personality disorder*") |
| S3 | (TI "personality disorder*") |
| S4 | ( (TI personality OR TI borderline ) AND ( TI disorder* OR TI dysfunction* OR TI pathology OR TI feature* OR TI trait* OR TI symptom*) ) |
| S5 | (MH "Musculoskeletal Diseases+") |
| S6 | (AB musculoskeletal) |
| S7 | (TI musculoskeletal) |
| S8 | (MH "Bone Density") |
| S9 | (AB bone*) |
| S10 | (TI bone*) |
| S11 | (MH "Fractures, Bone+") |
| S12 | (AB fracture*) |
| S13 | (TI fracture*) |
| S14 | (MH "Accidental Falls") |
| S15 | (AB fall*) |
| S16 | (TI fall*) |
| S17 | ( (TI physical OR TI medical OR TI chronic) AND ( TI illness* OR TI disease* OR TI condition* OR TI comorbidity OR TI problem) ) |
| S18 | ( (TI musculoskeletal* OR TI bone* OR TI fall* OR TI fracture* ) AND ( ( TI disease AND TI burden ) ) OR ( TI morbidity OR TI multimorbidity OR TI mortality OR TI disability* OR TI cost*) ) |
| S19 | S1 OR S2 OR S3 OR S4 |
| S20 | S5 OR S6 OR S7 OR S8 OR S9 OR S10 OR S11 OR S12 OR S13 OR S15 OR S16 OR S17 OR S18 |
| S21 | S19 AND S20 |

**Note**: Search modes = Boolean/Phrase. AB= search in abstract field; TI = search title field; MH = search in MeSH/Index Term field

### **CINAHL via EBSCOhost platform**

| Search line | Index/keyword/combinations |
| --- | --- |
| S1 | (MH "Personality Disorders+") |
| S2 | (AB "personality disorder*") |
| S3 | (TI "personality disorder*") |
| S4 | ( (TI personality OR TI borderline ) AND ( TI disorder* OR TI dysfunction* OR TI pathology OR TI feature* OR TI trait* OR TI symptom*) ) |
| S5 | (MH "Musculoskeletal Diseases+") |
| S6 | (AB musculoskeletal) |
| S7 | (TI musculoskeletal) |
| S8 | (MH "Bone Density") |
| S9 | (AB bone*) |
| S10 | (TI bone*) |
| S11 | (MH "Fractures+") |
| S12 | (AB fracture*) |
| S13 | (TI fracture*) |
| S14 | (MH "Accidental Falls") |
| S15 | (AB fall*) |
| S16 | (TI fall*) |
| S17 | ( (TI physical OR TI medical OR TI chronic) AND ( TI illness* OR TI disease* OR TI condition* OR TI comorbidity OR TI problem) ) |
| S18 | ( (TI musculoskeletal* OR TI bone* OR TI fall* OR TI fracture* ) AND ( ( TI disease AND TI burden ) ) OR ( TI morbidity OR TI multimorbidity OR TI mortality OR TI disability* OR TI cost*) ) |
| S19 | S1 OR S2 OR S3 OR S4 |
| S20 | S5 OR S6 OR S7 OR S8 OR S9 OR S10 OR S11 OR S12 OR S13 OR S15 OR S16 OR S17 OR S18 |
| S21 | S19 AND S20 |

**Note:** Search modes = Boolean/Phrase. AB= search in abstract field; TI = search title field; MH = Index Term field

### **PsyINFO via EBSCOhost platform**

| Search line | Index/keyword/combinations |
| --- | --- |
| S1 | (DE "Personality Disorders" OR DE "Antisocial Personality Disorder" OR DE "Avoidant Personality Disorder" OR DE "Borderline Personality Disorder" OR DE "Dependent Personality Disorder" OR DE "Histrionic Personality Disorder" OR DE "Narcissistic Personality Disorder" OR DE "Obsessive Compulsive Personality Disorder" OR DE "Paranoid Personality Disorder" OR DE "Passive Aggressive Personality Disorder" OR DE "Sadomasochistic Personality" OR DE "Schizoid Personality Disorder" OR DE "Schizotypal Personality Disorder") |
| S2 | (AB "personality disorder*") |
| S3 | (TI "personality disorder*") |
| S4 | ( (TI personality OR TI borderline ) AND (TI disorder* OR TI dysfunction* OR TI pathology OR TI feature* OR TI trait* OR TI symptom*) ) |
| S5 | (DE "Musculoskeletal Disorders" OR DE "Bone Disorders" OR DE "Bruxism" OR DE "Joint Disorders" OR DE "Muscular Disorders") |
| S6 | (AB musculoskeletal) |
| S7 | (TI musculoskeletal) |
| S8 | (AB bone*) |
| S9 | (TI bone*) |
| S10 | (AB fracture*) |
| S11 | (TI fracture*) |
| S12 | (DE "Falls") |
| S13 | (AB fall*) |
| S14 | (TI fall*) |
| S15 | ( (TI physical OR TI medical OR TI chronic) AND ( TI illness* OR TI disease* OR TI condition* OR TI comorbidity OR TI problem) ) |
| S16 | ( (TI musculoskeletal* OR TI bone* OR TI fall* OR TI fracture*) AND ( (TI disease AND TI burden) ) OR (TI morbidity OR TI multimorbidity OR TI mortality OR TI disability* OR TI cost*) ) |
| S17 | S1 OR S2 OR S3 OR S4 |
| S18 | S5 OR S6 OR S7 OR S8 OR S9 OR S10 OR S11 OR S12 OR S13 OR S14 OR S15 OR S16 |
| S19 | S17 AND S18 |

**Note:** Search modes = Boolean/Phrase. AB= search in abstract field; DE = Index Term field; TI = search title field

### **Adapted search for published grey literature**

| **Search ID** | **Grey Literature (i.e. government/peak body reports)**  **Google Advance Search—Find Pages with “all these words”; Narrow results by file type: PDF** |
| --- | --- |
| S1 | "personality disorder" AND "musculoskeletal diseases" OR "musculoskeletal disorders" AND "report" filetype:pdf |

# **Supplementary Table 2:** *Data extraction instrument*

| **Data items** |
| --- |
| ID |
| Author and year |
| Search type (database searching/Google search/snowballing) |
| Main aims |
| Study name |
| Context |
| Country |
| Study design |
| Sample size (including subgroups) |
| Mean age + SD/median + IQR/ or age group/range |
| Sex (including subgroups) |
| PD classification |
| PD instrument and administered |
| PD identified by expert/lay/self-report/other |
| MSD diagnosis |
| MSD identified by expert/lay/self-report/other |
| Measure of PD + MSDs burden |
| Main results |

# **Supplementary Table 3:** *General characteristics of the 57 included articles (in alphabetic order of citation)*

| Citation & country | Study design (follow-up/data collection period) | Study population; sample size (*n*) | Mean age  (SD)/median (IQR)/age range | Sex % | Assessment of PD | Assessment of MSD | Burden concept | Relevant statistical approach |
| --- | --- | --- | --- | --- | --- | --- | --- | --- |
| Attademo & Bernardini, 2018 | Narrative review examining the frequency of PD among patients with fibromyalgia | na | na | na | Inclusion criteria nr | Inclusion criteria nr | na | na |
| Braden & Sullivan, 2008  USA | Cross-sectional  (February 2001–April 2003) | Community-based respondents enrolled in the NCS-R  N: 5,692 | Aged 18+ | F: 58.6%  (with lifetime self-reported pain)  F: 46.6% (without lifetime self-reported pain) | ICD-10  Adapted IPDE screener using borderline PD and antisocial items (self-report) | Chronic back/neck pain (self-report) | na | Descriptive statistics  Logistic regression analysis |
| Breckenridge & Clark, 2003  USA | Retrospective cohort  (August 2001 –February 2002) | Patients attending the Stanford University and  the Veterans Affairs Palo Alto Health Care System  N: 200  “N” group: 100 (received NSAIDs)  “O” group: 100 (received opioid drugs) | N: 61.8 (11.7)  O: 61.5 (13.0) | F: 5% (N)  F: 6% (O) | ICD-9  Chart review | Chart review of grouped backache/lumbago, postlaminectomy syndrome/ lumbosacral neuritis/ lumbosacral spondylosis without myelopathy/ displacement of lumbar disk/degeneration of lumbar or lumbosacral  disk/lumbar spinal stenosis (according to ICD-9 codes) | Morbidity | Descriptive statistics  Multivariate logistic regression analysis |
| Brede et al., 2011  USA | Cross-sectional  (dates nr) | Patients entering the PRIDE functional restoration program  N: 551 | 47.2 (9.9) | M: 52% | DSM-IV  SCID-II (expert) | Grouped musculoskeletal disorders involving pain/injury of cervical/ thoracic/lumbar extremity/multiple spinal/multiple musculoskeletal with at least one spinal (expert diagnosis) | Patient-reported outcome | Descriptive statistics  ANOVAs |
| Campbell et al., 2015  Australia | Cross-sectional (baseline; dates ns) | Participants with chronic non-cancer pain enrolled in the POINT study recruited through community pharmacies N: 978 | 57.5 (13.6) | M: 44.7% | ICD-10  Adapted IPDE screener using borderline PD items (self-report) | Arthritis, chronic back/neck pain, and fibromyalgia (self-report) | Morbidity | Descriptive statistics  Logistic regression analysis |
| Dersh et al., 2002  USA | Cross-sectional  (January 1993– December 1998) | Patients entering the PRIDE functional restoration program  N: 1,595 | 42.1 (9.6) | F: 41.9%  M: 58.1% | DSM-IV  SCID-II (expert) | Grouped musculoskeletal/spina disorders grouped according to pain/injury site: lumbar spine, cervical spine, multiple spine areas, upper extremity neuropathic, upper extremity nonneuropathic, and three or more (polymorphous) musculoskeletal areas (expert diagnosis) | na | Descriptive statistics |
| Dersh et al., 2006  USA | Cross-sectional  (dates nr) | Patients entering the PRIDE functional restoration program  N: 1,323 | 41.9 (9.6) | F: 38.3%  M: 61.7% | DSM-IV  SCID-II (expert) | Grouped spinal disorders according to pain/injury site: cervical and/or thoracic, lumbar, and cervical/thoracic and lumbar (expert diagnosis) | na | Descriptive statistics |
| Dersh et al., 2007  USA | Prospective cohort  (one-year follow-up; dates nr) | Patients before and after receiving treatment in the PRIDE functional restoration program  N: 1,323 | 41.9 (9.6) | F: 38.3%  M: 61.7% | DSM-IV  SCID-II (expert) | Grouped musculoskeletal/spinal disorders according to pain/injury site: cervical and/or thoracic, lumbar, multiple spinal, multiple musculoskeletal with at least one spinal (expert diagnosis) | Clinician-reported outcome  Work-related outcome | Descriptive statistics  Logistic regression analysis |
| Dixon-Gordon et al., 2015 | Systematic (narrative) review examining associations between PD and sleep disturbance, obesity, pain conditions, and other chronic health conditions | na | na | na | DSM-III-5 | Health-related outcomes | na | na |
| Dixon-Gordon et al., 2018 | Updated narrative review to summarise research on associations between PD and health conditions | na | na | na | Inclusion criteria nr | Inclusion criteria nr | na | na |
| Doering, 2019 | Narrative review to examine epidemiological studies on borderline PD and comorbidity with somatic illness | na | na | na | Inclusion criteria nr | Inclusion criteria nr | na | na |
| Dokucu & Cloninger, 2019 | Narrative review on studies conducted in the decade 2009-2019 on associations between PD and medical conditions | na | na | na | Inclusion criteria nr | Inclusion criteria nr | na | na |
| Douzenis et al., 2012 | Narrative review to examine the medical comorbidity of Cluster B PD | na | na | na | Inclusion criteria nr | Inclusion criteria nr | na | na |
| El-Gabalawy et al., 2010  USA | Cross-sectional  (Wave 2 2004-2005) | Wave 2 NESARC participants  N: 34,653 | Aged 20+ | F: 52.1%  M: 47.9% | DSM-IV  AUDADIS-IV (lay interviewer) | Arthritis (self-report) | na | Descriptive statistics  Logistic regression analysis  Regression analysis |
| El-Gabalawy et al., 2014  USA | Prospective cohort; 2-year follow-up  (Wave 1 2001–2002 and Wave 2 2004–2005) | Wave 1 and 2 NESARC participants aged 55+  N: 10,409 | Aged 55+ | F: 55.4%  M: 44.6% | DSM-IV  AUDADIS-IV (lay interviewer) | Arthritis (self-report) | na | Descriptive statistics  Logistic regression analysis |
| Ericsson et al., 2002  Sweden | Prospective cohort  (two-and-a-half years’ follow-up; dates nr) | Chronic pain patients attending a National Social Insurance Hospital  N: 184 | 43.4 (10.8) | F 72.8% | DSM-III-R  SCID-II Screen (self-report) | Grouped chronic pain at multiple sites/localized neck/back/extremity pain identified from a review of insurance records | Disability indicator | Descriptive statistics  Logistic regression analysis |
| Fietta et al., 2007  Italy | Narrative review to examine the frequency of psychiatric disorders in patients with fibromyalgia | na | na | na | Inclusion criteria nr | Inclusion criteria nr | na | na |
| Fishbain et al., 2007  USA | Cross-sectional  (March 1991–March 1993) | Chronic pain patients attending the University of Miami Comprehensive Pain Center  N: 221 | 41.1 (10.0) | F: 42%  M: 58% | DSM-III-R  Clinical impression/ flowchart | Chronic low back pain (self-reported/presenting problem) | na | Descriptive statistics  Logistic regression analysis |
| Fok et al., 2019  UK | Retrospective cohort  (6-year observation period between 1 April 2007–31 March 2013) | Patients receiving care from the SLaM service  N: 7,677 | 36.32 (14.69) | F: 55.75%  M: 44.08% | ICD-10 PD Diagnoses searched using  CRIS at SLaM and GATE language processing software from case notes/correspondence | ICD-10 general hospital admission/discharge diagnoses using linked HES data | Hospital admission | Standardised admission ratios |
| Frankenburg & Zanarini, 2004  USA | Prospective cohort  (6-year follow-up; June 1992–December 2001) | Patients enrolled in the MSAD study  N:264  Ever remitted: 200  Never remitted: 64 | Ever remitted: 32.5 (5.8)  Never remitted: 34.5 (5.8) | F: 80.0% (ever remitted)  F: 82.8% (never remitted) | DSM-III-R  DIB-R (expert) | Osteoarthritis and chronic back pain (expert diagnosis) | Clinician-reported outcome | Descriptive statistics  Logistic regression analysis |
| Frankenburg & Zanarini, 2006a  USA | Prospective cohort  (6-year follow-up; June 1992–December 2001) | Patients enrolled in the MSAD study  N: 264  Borderline PD with obesity: 74 Borderline PD without obesity: 190 | Borderline PD with obesity 35.0 (6.1)  Borderline PD without obesity: 32.2 (5.6) | F: 87.8% (borderline PD with obesity)  F: 77.9 (border line PD without obesity) | DSM-III-R  DIB-R (expert) | Osteoarthritis and chronic back pain (expert diagnosis) | na | Descriptive statistics  Logistic regression analysis |
| Frankenburg & Zanarini, 2006b  USA | Narrative review to examine PD and medical comorbidity | NA | NA | NA | Inclusion criteria nr | Inclusion criteria nr | na | NA |
| Frankenburg et al., 2014  USA | Prospective cohort (July 1998–December 2010) | Patients enrolled in the MSAD study  N: 264 | 33.0 (SD=5.8) | F: 80.7% | DSM-III-R  DIB-R (expert) | Osteoarthritis, chronic back pain, and fibromyalgia (expert diagnosis) | Morbidity | Descriptive statistics  Logistic regression analysis |
| Fu et al., 2015  USA | Cross-sectional (dates nr) | Patients attending an outpatient rheumatology office  N: 48 | 49.3 (nr) | F: 95.8  M: 4.2% | DSM-IV  PDQ-4 (self-report) | Rheumatology department record review  fibromyalgia according to ACR criteria | na | Descriptive statistics |
| Gatchel et al., 1994  USA | Prospective cohort (one-year follow-up; dates nr) | Patients before and after receiving treatment in the PRIDE functional restoration program  N: 152  Return-to-work group: 129  No return-to-work group: 23 | Return-to-work: 35.7 (8.9)  No return-to-work: 37.1 (7.2) | F: 35% (return-to-work)  M: 65% (return-to-work)  F: 43% (no-return-to-work)  M: 57% (non-return-to-work) | DSM-IV  SCID-II (expert) | Chronic low back pain including degenerative disc disease, lumbar radicular syndrome, postoperative epidural fibrosis, segmental instability, and non-specific back pain (expert diagnosis) | Work-related outcomes | Descriptive statistics |
| Gatchel et al., 2006  USA | Prospective cohort (one-year follow-up; dates nr) | Patients entering the PRIDE functional restoration program  N: 1,489 | 42.3 (9.7) | M: 57.2% | DSM-IV  SCID-II (expert) | Grouped musculoskeletal/spinal disorders (expert diagnosis) | Work-related outcomes | Descriptive statistics  ANOVAs |
| Gerhardt et al., 2011  Germany | Cross-sectional (dates nr) | Population-based respondents of a postal survey of back pain by the GBPRN  N: 110 | 18-74 | F:57% | DSM-IV  SCID-II (nr) | Non-specific chronic back pain (Self-report; expert verified) | na | Descriptive statistics  Logistic regression analysis |
| Goldstein et al., 2008  USA | Cross-sectional  (Wave 1 2001–2002) | Wave 1 NESARC participants  N: 43,093 | 48 (13.3) | nr | DSM-IV  AUDADIS-IV (lay interviewer) | Arthritis (self-report) | na | Descriptive statistics  Logistic regression analysis |
| Gumà-Uriel et al., 2016  Spain | Cross-sectional  (2005–2008) | Patients enrolled in the FibroQoL study, a psychoeducational programme for fibromyalgia  N: 157 | 18–75 | F: 98.1% M: 1.9% | DSM-IV  IPDE Screener (self-report) | Identified patients with fibromyalgia according to ARC criteria from a database at the Viladecans Hospital | Patient-reported outcome  Financial costs | Descriptive statistics  ANOVA  Multivariate regression analysis |
| Howard et al., 2009  USA | Prospective cohort  (January 1996–December 2004) | Patients before and after receiving treatment in the PRIDE functional restoration program  N: 3,052  “Completer” group: 2,367  “non-completer” group: 685 | Completer: 45.1 (9.62)  Non-completer: 45.2 (10.48) | M: 53.7%  (completer)  M: 53.6% (non-completer) | DSM-IV  SCID-II (expert) | Musculoskeletal/spinal disorders according to pain/injury sites:  cervical, thoracic/lumbar, multiple spinal, multiple musculoskeletal, upper extremity, lower extremity  upper and lower but no spine (expert diagnosis) | Clinician-reported outcome | Descriptive statistics  Logistic regression analysis |
| Howard, 2010  USA | Cross-sectional  (January 1997–December 2010) | Patients entering the PRIDE functional restoration program  N: 3,492 | *Varies depending on subgroup examined | *Varies depending on subgroup examined | DSM-IV  SCID-II (expert) | Grouped musculoskeletal/spinal disorders grouped according to pain/injury site: lumbar spine, cervical spine, multiple spine areas, upper extremity neuropathic, upper extremity nonneuropathic, and three or more (polymorphous) musculoskeletal areas (expert diagnosis) | na | Descriptive statistics |
| Kahl et al., 2005  Germany | Cross-sectional^[[1]](#footnote-1)^ (dates nr) | Patients attending a Specialised unit for the treatment of borderline PD  N: 38  Borderline PD alone: 16  Borderline PD +ever MDD: 12  Borderline PD +current MDD: 10  Controls^1^: 20 | Borderline PD alone = 25.9 (5.0); Borderline PD+MDD = 31.8 (6.5); controls^1^: 24.2 (5.9) | F: 100% | DSM-IV  SCID-II (expert) | BMD measured using dual-energy X-ray absorptiometry at the lumbar spine, right femur, left femur, and the forearm of the nondominant hand Osteopenia defined as a T-score ≤–1 | na | Descriptive statistics  Z-scores of BMD  T scores for fracture risk |
| Kahl et al., 2006  Germany | Cross-sectional (dates nr) | Patients attending a Specialised unit for the treatment of borderline PD  MDD30: 12  MDD43: 12  Borderline PD+MDD: 23  Borderline PD alone: 16 | MDD: 20–51 years; MDD30: 30; MDD43: 42.9; Borderline PD+MDD = 18–43 years; Borderline PD alone: 19-34, | F: 100% | DSM-IV  SCID-II (expert) | BMD measured using dual-energy X-ray absorptiometry at the lumbar spine, right femur, left femur, and the forearm of the nondominant hand  Osteopenia defined as a T-score ≤–1 | na | Descriptive statistics  Z-scores of BMD  T scores for fracture risk |
| Kayhan et al., 2016  Turkey | Case-control (dates nr) | Patients with fibromyalgia attending the Outpatient Physical Therapy Unit  of Mevlana University  N: 190  Patient group: 96  Healthy group: 94 | 37.75 (6.24)  Patient: 38.27 (6.18) Healthy: 37.23 (6.29) | F: 100% | DSM-IV  SCID-II (expert) | Fibromyalgia according to ARC criteria (expert diagnosis) | na | Descriptive statistics  Logistic regression analysis |
| Keuroghlian et al., 2013  USA | Prospective cohort  (6–16-year follow-ups) | Patients enrolled in the MSAD study  N:264  Ever recovered: 134 Never recovered: 97 | 33.0 (SD=5.9) | F: 80.7% | DSM-III-R  DIB-R (expert) | Osteoarthritis and chronic back pain (expert diagnosis) | Clinician-reported outcome | Descriptive statistics  Logistic regression analysis |
| Linder et al., 2009  Sweden | Cross-sectional  (dates nr) | Patients referred by an insurance office to the Diagnostic Centre at the Karolinska Hospital who were long-term sick leavers  N: 416  Fibromyalgia: 92  Myalgia group: 44  Spine/joints: 111  Depression: 169 | Fibromyalgia: 45.6 (10.2)  Myalgia: 44.4 (8.1)  Spine/joints: 46.4 (8.2)  Depression: 46.5 (9.5) | F: 100% | DSM-IV  SCID-II (expert) | Fibromyalgia, myalgia, and diseases of spine/joints according to ICD-10 criteria (expert diagnosis) | na | Descriptive statistics |
| Long et al., 1988  USA | Cross-sectional  (1979–1981) | Patients who were treated for chronic back pain at the Johns Hopkins Pain Treatment Program  N: 78 | 19–67 | F: 66.1%  M: 58.1% | DSM-III  Clinical impression/  collateral | Chronic low back pain according to review of medical history, imaging and operative records at discharge | na | Descriptive statistics |
| López-Ruiz et al., 2019  Spain | Case-control | Patients attending the Rheumatology Departments of the Hospital del Mar and Hospital CIMA-Sanitas in Barcelona  OA-CS group: 19  OA-noCS group: 41  Fibromyalgia group: 47  Control group: 26 | OA-CS: 66.37 (8.77)  OA-noCS: 66.8 (7.39)  Fibromyalgia: 46.47 (7.92)  Control: 51.56 (11.41) | F: 84.2% (OA-CS)  F: 65.9% (OA-noCS)  F: 100% (fibromyalgia)  F: 59.3% (control) | DSM-IV  MCMI-III (self-report) | Osteoarthritis (with and without CS) and fibromyalgia according to ARC criteria (expert diagnosis) | na | Descriptive statistics  Logistic regression analysis |
| Marcenaro et al., 1999  Italy | Cross-sectional | In- and outpatients receiving treatment at a rheumatology department  N: 15 | 54 (12.8) | F: 80%  M: 20% | DSM-III-R  SCID-II (nr) | Rheumatoid arthritis (expert diagnosis) | na | Descriptive statistics |
| McWilliams et al., 2008  USA | Cross-sectional  (Wave 1 2001–2002) | Wave 1 NESARC participants  N: 43,093 | Aged 18+ | nr | DSM-IV  AUDADIS-IV (lay interviewer) | Arthritis (self-report) | na | Descriptive statistics  Logistic regression analysis |
| McWilliams & Higgins, 2013  USA | Cross-sectional (NCS Part II 2001–2002) | Community-based respondents enrolled in Part II of the NCS-R  N: 5,692 | Aged 18+ | nr | ICD-10  Adapted IPDE screener using borderline PD items (self-report) | Arthritis, chronic back/neck pain (self-report) | na | Descriptive statistics  Logistic regression analysis |
| Olssøn & Dahl, 2009  Norway | Case-control  (May 2000–September 2001) | Community-based respondents to the HUBRO study health  Survey  N: 2,214  Cases: 369  Controls: 1,845 | 30+ | F: 48%  M: 52% | DSM-IV  Iowa Personality Disorder Screen | Musculoskeletal pain and fibromyalgia (self-reported) | na | Descriptive statistics  Logistic regression analysis |
| Olssøn & Dahl, 2012  Norway | Case-control  (May 2000–September 2001) | Community-based respondents to the HUBRO study health  Survey  Cases:280  Controls: 1,400 | 30+ | F: 65%  M:35% | DSM-IV  Avoidant PD items of the Iowa Personality Disorder Screen | Muscular pain (self-reported) | na | Descriptive statistics  Logistic regression analysis |
| Perish, 2012  USA | Prospective cohort  (November 2003–January 2009) | Patients before and after receiving treatment in the ALBP at The University of Texas Southwestern Medical Center  N: 53  Completers group: 30  Non-completers group = 23 | 41.58 (11.19)  19 to 63 | F: 49.1%  M: 50.9% | DSM-IV  SCID-II (expert) | Acute low back pain (expert diagnosis) | Clinician-reported outcome | Descriptive statistics |
| Polatin et al., 1993  USA | Cross-sectional (dates; nr) | Patients entering the PRIDE functional restoration program  N: 200 | nr | F: 33% | DSM-III-R  SCID-II (expert) | Chronic low back pain (expert diagnosis) | na | Descriptive statistics |
| Powers & Oltmanns, 2013  USA | Cross-sectional (dates nr) | Community-based residents aged 55–64 years enrolled in the SPAN  N: 1,051 | 59.4 (2.7) | F: 53% | SIDP-IV (trained interviewers) | Arthritis (self-reported) | na | Descriptive statistics  Logistic regression analysis  Mediation analysis |
| Quirk et al., 2015  USA | Cross-sectional  (pooled Wave 1 2001–2002; Wave 2 2004–2005) | Wave I and 2 NESARC participants  N: 34,653 | Aged 20+ | F: 52.1%  M: 47.9% | DSM-IV  AUDADIS-IV (lay interviewer) | Arthritis (self-reported) | na | Descriptive statistics  Logistic regression analysis |
| Quirk et al., 2016  Australia | Cross-sectional  (2011–2014) | Community-based women enrolled in the GOS in south-eastern Australia  N: 765 | 56.8 (42.7–68.9 | F: 100% | DSM-5  SCID-II (trained interviewer) | Arthritis (self-reported) | na | Descriptive statistics  Logistic regression analysis |
| Quirk et al., 2016 | Narrative review to examine population-based associations between PD and physical comorbidities and service utilization | NA | NA | NA | Inclusion criteria nr | Inclusion criteria nr | na | NA |
| Russek et al., 2015  USA | Cross-sectional (2010–2012) | Survey respondents accessing the National Fibromyalgia Association website  N: 1,125 | Median range 40–49 | F: 97.6% | DSM-IV  Self-report questionnaire based on criteria for OCPD | Fibromyalgia (self-reported) | na | Descriptive statistics |
| Sansone et al., 2009  USA | Cross-sectional (dates nr) | Admission to a sub-acute detoxification unit for opioid dependence, in which buprenorphine is the standardised treatment  N: 111 | 18 to 59 years (M‑32.80, SD‑9.04) | F: 46.5%  M: 53.5% | DSM-IV  PDQ-4 (self-report) | Rheumatoid arthritis and fibromyalgia (self-report) | na | Descriptive statistics |
| Sansone & Sansone, 2012 | Narrative review to examine the frequency of borderline PD in chronic pain populations and relevant associations | na | na | na | nr | nr | na | na |
| Schubert et al., 1995  USA | Cross-sectional (17-month period) | Consecutive admissions to a psychiatry ward at Metro Health Medical  Center, Cleveland, Ohio  N: 532  Psychiatric dx without physical dx: 222  Psychiatric dx + physical dx: 310 | Total: mean age range 30-46 Psychiatric dx no physical dx: 33.2 (10.5) Psychiatric dx + physical dx: 43.0 (15.3) | F: 66% | ICD-9  Psychiatrist diagnosis | Diagnoses of musculosystem and connective tissue diseases ascertained from hospital records according to ICD-9 | Hospital admission | Descriptive statistics  ANOVA |
| Thieme et al., 2004  Germany | Cross-sectional (dates nr) | Patients attending a rheumatologic outpatient department and Hospital  for Rheumatic Disorders at Berlin-Buch  N: 115 | 48.17 (10.32) | F: 100% | DSM-IV  SCID-II (expert) | Fibromyalgia according to ARC criteria (expert diagnosis) | na | Descriptive statistics |
| Uguz et al., 2010  Turkey | Case-control (dates nr) | Patients attending Rheumatology Outpatient Clinic at a University hospital  N: 103 cases  N: 83 controls | nr | nr | DSM-III-R  SCID-II (expert) | Fibromyalgia according to ARC criteria (expert diagnosis) | na | Descriptive statistics |
| Uguz et al., 2015  Turkey | Case-control (dates nr) | Patients attending a Rheumatology Outpatient Clinic of the Research and Training Hospital of Necmettin Erbakan University  With PD: 30  Without PD: 112  Controls: 60 | 42.64 (10.64) | F: 93.1% | DSM-III-R  SCID-II (expert) | Fibromyalgia according to ARC criteria (expert diagnosis) | Patient-reported outcome | Descriptive statistics  Spearman’s correlation test |
| Williams et al., 2020  Australia | Cross-sectional  (2011–2014) | Community-based women enrolled in the GOS in south-eastern Australia (2011-2014)  N: 696 | 56.8 (42.7–68.9) | F: 100% | DSM-5  SCID-II (trained interviewer) | Areal BMD (g/cm2) was measured at the posterior–  anterior spine, femoral neck (hip), and total body including head using dual-energy X-ray absorptiometry Osteoporosis was determined by a BMD T-score of <-2.5 at the spine and/or hip | na | Descriptive statistics  T-score  Regression analysis |

*Note:* ALBP=Acute Low Back Pain Program; ACR=American College of Rheumatology; AUDADIS-IV=Alcohol Use Disorder and Associated Disabilities Interview Schedule-IV; BMD=Bone mineral density; CRIS=Clinical Record Interactive Search; CS=Central sensitisation; DIB-R=Diagnostic Interview for Borderlines-Revised; DSM=Diagnostic and Statistical Manual of Mental Disorders; Dx=diagnosis; F=female; GATE=Generalised Architecture for Text Engineering; GBPRN=German Back Pain Research Network; GOS=Geelong Osteoporosis Study; HES=Hospital Episodes Statistics; HUBRO=The Oslo Health Study; ICD=International Classification of Diseases and Related Health Problems; IPDE=International Personality Disorder Examination; MCMI= Millon Clinical Multiaxial Inventory; M=male; MDD=Major depressive disorder; MSAD=McLean Study of Adult Development; MSD=Musculoskeletal disorders; na=not applicable; NCS-R=National Comorbidity Survey-Revised; NESARC=National Epidemiological Survey on Alcohol and Related Conditions; nr=not reported; NSAID=Nonsteroidal anti-inflammatory drug; OA=osteoarthritis; PA=Posterior-anterior; PD=Personality disorder; PDQ-4=Personality Diagnostic Questionnaire-4; POINT=Pain and Opioids IN Treatment; PRIDE=Productive Rehabilitation Institute of Dallas for Ergonomics; QoL=Quality of life; SCID-II=Structured Clinical Interview for DSM Axis II Personality Disorders; SLaM=South London and Maudsley NHS Foundation Trust; SIDP-IV=Structured Interview for DSM-IV Personality; SPAN=St. Louis Personality and Aging Network

1. In this study, controls were not measured on outcome of interest for this review (BMD) [↑](#footnote-ref-1)
